# CpG Site-Specific Methylation-Modulated Divergent Expression of *PRSS3* Transcript Variants Facilitates Nongenetic Intratumor Heterogeneity in Human Hepatocellular Carcinoma

**DOI:** 10.3389/fonc.2022.831268

**Published:** 2022-04-11

**Authors:** Shuye Lin, Hanli Xu, Mengdi Pang, Xiaomeng Zhou, Yuanming Pan, Lishu Zhang, Xin Guan, Xiaoyue Wang, Bonan Lin, Rongmeng Tian, Keqiang Chen, Xiaochen Zhang, Zijiang Yang, Fengmin Ji, Yingying Huang, Wu Wei, Wanghua Gong, Jianke Ren, Ji Ming Wang, Mingzhou Guo, Jiaqiang Huang

**Affiliations:** ^1^ Cancer Research Center, Beijing Chest Hospital, Capital Medical University, Beijing Tuberculosis and Thoracic Tumor Institute, Beijing, China; ^2^ College of Life Sciences & Bioengineering, Beijing Jiaotong University, Beijing, China; ^3^ Department of Gastroenterology and Hepatology, Chinese People’s Liberation Army of China (PLA) General Hospital, Beijing, China; ^4^ Laboratory of Cancer Immunometabolism, Center for Cancer Research, National Cancer Institute, Frederick, MD, United States; ^5^ Chinese Academy of Sciences (CAS) Key Laboratory of Computational Biology, Shanghai Institute of Nutrition and Health, University of Chinese Academy of Sciences, Chinese Academy of Sciences, Shanghai, China; ^6^ Basic Research Program, Leidos Biomedical Research, Inc., Frederick, MD, United States

**Keywords:** liver cancer, hepatocellular carcinoma, *PRSS3*, transcript variant, intratumor heterogeneity, CpG methylation, epigenetics, biomarker

## Abstract

**Background:**

Hepatocellular carcinoma (HCC) is one of the most lethal human tumors with extensive intratumor heterogeneity (ITH). Serine protease 3 (PRSS3) is an indispensable member of the trypsin family and has been implicated in the pathogenesis of several malignancies, including HCC. However, the paradoxical effects of *PRSS3* on carcinogenesis due to an unclear molecular basis impede the utilization of its biomarker potential. We hereby explored the contribution of *PRSS3* transcripts to tumor functional heterogeneity by systematically dissecting the expression of four known splice variants of *PRSS3* (*PRSS3-SVs, V1~V4*) and their functional relevance to HCC.

**Methods:**

The expression and DNA methylation of *PRSS3* transcripts and their associated clinical relevance in HCC were analyzed using several publicly available datasets and validated using qPCR-based assays. Functional experiments were performed in gain- and loss-of-function cell models, in which *PRSS3* transcript constructs were separately transfected after deleting *PRSS3* expression by CRISPR/Cas9 editing.

**Results:**

*PRSS3* was aberrantly differentially expressed toward bipolarity from very low (*PRSS3^Low^*) to very high (*PRSS3^High^*) expression across HCC cell lines and tissues. This was attributable to the disruption of *PRSS3-SVs*, in which *PRSS3-V2* and/or *PRSS3-V1* were dominant transcripts leading to *PRSS3* expression, whereas *PRSS3-V3* and *-V4* were rarely or minimally expressed. The expression of *PRSS3-V2* or *-V1* was inversely associated with site-specific CpG methylation at the *PRSS3* promoter region that distinguished HCC cells and tissues phenotypically between hypermethylated low-expression (m*PRSS3-SV^Low^*) and hypomethylated high-expression (um*PRSS3-SV^High^*) groups. *PRSS3-SVs* displayed distinct functions from oncogenic *PRSS3-V2* to tumor-suppressive *PRSS3-V1*, *-V3* or *PRSS3-V4* in HCC cells. Clinically, aberrant expression of *PRSS3-SVs* was translated into divergent relevance in patients with HCC, in which significant epigenetic downregulation of *PRSS3-V2* was seen in early HCC and was associated with favorable patient outcome.

**Conclusions:**

These results provide the first evidence for the transcriptional and functional characterization of *PRSS3* transcripts in HCC. Aberrant expression of divergent *PRSS3-SVs* disrupted by site-specific CpG methylation may integrate the effects of oncogenic *PRSS3-V2* and tumor-suppressive *PRSS3-V1*, resulting in the molecular diversity and functional plasticity of *PRSS3* in HCC. Dysregulated expression of *PRSS3-V2* by site-specific CpG methylation may have potential diagnostic value for patients with early HCC.

## Introduction

Human primary liver cancer is one of the most lethal tumors with a dismal prognosis, featuring extensive intratumor heterogeneity (ITH) and aggressiveness in the context of genetic and epigenetic aberrations (1–5). Liver hepatocellular carcinoma (HCC or LIHC) accounts for approximately 75-85% of all primary liver cancers. Most HCCs (>90%) develop from chronic inflammation-induced liver cirrhosis contributed by multiple risk factors, such as hepatitis viruses, alcohol consumption, and nonalcoholic fatty liver disease, which trigger the molecular complexity of ITH, increasing HCC phenotypic diversity and therapeutic resistance (3, 5). Regardless of the many approaches developed for the management of HCC in the past decade, its incidence and mortality rate continue to increase worldwide (5).

Large-scale bioinformatics datasets generated with next-generation sequencing technologies reveal a comprehensive landscape of genomic and epigenetic heterogeneity among HCC cell lines and tissue specimens (1, 2, 4, 6, 7). These studies offer invaluable insight into the molecular basis of ITH to categorize HCC into proliferative and nonproliferative subclasses in favor of integrative molecular monitoring of malignant transformation and management of HCC. However, aside from most genetic alterations occurring in passenger genes that may be associated with aging and pollution, most genetic variants, such as driver mutations in *TP53, TERT* and *CTNNB1* detected in HCCs, are not clinically relevant or are not potentially targetable for the existing drugs (3). This gives rise to a growing drive to integrate nongenetic variations into ITH and to distinguish between functional and nonfunctional ITH (7, 8). PremRNA alternative splicing (AS), as a key co and posttranscriptional process, drives nongenetic phenotypic heterogeneity, the disruption of which generates aberrant transcript variants or splice variants (*SVs*) that contribute to ITH and functional divergence and are thus functionally important to carcinogenesis and oncotherapeutic resistance (9–12).

Proteases play critical roles in multiple biological processes and are associated with a wide variety of pathological conditions, including carcinogenesis (13). As a group of trypsin-family serine proteases, human trypsinogen gene, protease serine 3 (*PRSS3*), encodes PRSS3, also called mesotrypsinogen (MTG) (14–16). *PRSS3* possesses four experimentally validated *SVs*, referred to as trypsinogen transcript variants 1, 2, 3, and 4 (*PRSS3-V1*, *-V2*, *-V3* and *-V4*), encoding PRSS3 isoform 1 (also known as brain form or trypsinogen 4, TRY4) (15, 17), PRSS3-2 (form C or MTG) (14, 18), PRSS3-3 (form B or trypsinogen IV) (19), and PRSS3-4 (new form or trypsinogen 5), respectively (20). In addition to PRSS1 and PRSS2, as the major digestive enzymes in the pancreas, PRSS3 is a minor constituent trypsin isoform but is physiologically critical due to its resistance to common trypsin inhibitors (13, 14, 16). PRSS3 has long been implicated in the pathogenesis of several malignancies and is therefore a promising biomarker and potential therapeutic target for cancer (21–31). However, the functional roles associated with the expression of *PRSS3* in cancer development are debatable. On the one hand, PRSS3 was shown to be upregulated in association with cancer metastasis, recurrence and poor prognosis (21–24, 26–31). However, on the other hand, *PRSS3* was suggested to be a tumor suppressor gene due to epigenetic silencing (32–36). Although the evidence supports the dual roles of proteases in carcinogenesis depending on cellular sources and the cancer microenvironment (9, 12, 13, 22, 23, 26, 34–36), the underlying molecular basis of PRSS3 for its pro- and antitumorigenic roles shown in different cancer types, even reported in the same type of cancer, such as in esophageal adenocarcinoma (24, 32), lung cancer (29, 35) and liver cancer (21, 36), remains elusive, causing many miscellaneous aliases to *PRSS3* to impact its potential target-therapeutic applications (1, 12, 13, 23, 25, 36).

While *SVs* have emerged as new candidates for diagnostic and prognostic biomarkers and therapeutic targets (9, 10), the expression and function of *PRSS3-SVs* in cancer development have never been systematically addressed. Here, we hypothesized that the molecular basis of *PRSS3* exerts dual roles attributable to its different transcripts. We thereby investigated the functional expression and epigenetic alteration of *PRSS3-SVs* in relation to HCC heterogeneity. We found divergent expression of *PRSS3-SVs* in HCC, which were epigenetically dysregulated by site-specific abnormal CpG methylation. We also observed different functionalities and clinical relevance of *PRSS3-SVs*. Therefore, epigenetic dysregulation of the expression of *PRSS3-SVs* may integrate the molecular basis of *PRSS3* to exert divergent effects on hepatocarcinogenesis.

## Materials and Methods

### Data Collection

The datasets used for this study are publicly available on the following websites: the Cancer Model Repository (LIMORE) (https://www.picb.ac.cn/limore/home) (6); the Cancer Genome Atlas (TCGA, https://www.cancer.gov/) (38); the Gene Expression Profiling Interactive Analysis (GEPIA, http://gepia.cancer-pku.cn/) (37); the Cancer Cell Line Encyclopedia (CCLE, http://www.broadinstitute.org/ccle) (39); the Cancer Dependency Map (DepMap, https://depmap.org/portal/, DepMap Public 20Q3) (40); and the Broad Genome Data Analysis Center (http://gdac.broadinstitute.org) (41). The expression of PRSS3 protein was analyzed using data obtained from the Clinical Proteomic Tumor Analysis Consortium (CPTAC) Confirmatory/Discovery dataset (http:ualcan.path.uab.edu) (42).

### Cell Lines

Human HCC cell lines, including well differentiated (HepG2 and Huh7) and poorly differentiated (SK-Hep-1, SMMC-7721 and LM3) cell lines, were purchased from Cellcook Biotech Co. (Guangzhou, China) and authenticated by STR profiling (Additional files). The cell lines were grown in DMEM (Gibco, Life Technologies, USA) with 10% fetal bovine serum (Gibco, USA), penicillin/streptomycin and glutamine as described previously (36, 43). *TransSafe™* Mycoplasma Prevention Reagent was used to prevent mycoplasma contamination (TransGene, China). The cells were split to low density (30% confluence) overnight culture and were then treated with 5 μM 5-aza-2’-deoxycytidine (5-aza-CR) (Sigma–Aldrich, USA) for 96 hours, with the medium exchanged every 24 hours.

### Cell Line Construction

The establishment of stable cell lines with *PRSS3-V1* overexpression was described previously (36). The OmicsLink™ Expression clones of *PRSS3-V2, -V3* and *-V4* were purchased from GeneCopoeia (Rockville, MD, USA) (**Table S6**). The CRISPR/Cas9 bivector lentivirus was custom ordered from GeneChem (Shanghai, China). The sgRNA was GGCACTGAGTGCCTCATCTC. Genomic deletion of *PRSS3* transcripts (*PRSS3^KO^*) by targeting the common exon 5-8 region in *PRSS3^High^* Huh7 cells was performed using the CRISPR/Cas9 system. Puromycin (Puro) (2 μg/ml) was used for selection of the transduced cells. *PRSS3^KO^* Huh7 cells were transfected with the *PRSS3-V1* to -*V4* constructs to establish stable re-expression of *PRSS3* transcripts dubbed the *PRSS3^KO+V^* cell model. Transfection was performed using Lipofectamine 2000 (Invitrogen, USA) following the manufacturer’s instructions. Stable cell lines with *PRSS3-V2, -V3* or *-V4* were selected using 0.5 mg/ml G418 (Invitrogen) for 2 weeks.

### Cell Viability

HepG2, SK-Hep-1 and Huh7 cells were seeded into 96-well plates at 2 × 10^3^ cells/well. Cell viability was measured every day by using a 3-(4,5-dimethylthiazol-2-yl)-2,5-diphenyltetrazolium bromide (MTT) assay kit (KeyGEN Biotech, China). The absorbance at 490 nm was detected using a microplate reader (Thermo Multiskan MK3, Thermo Fisher Scientific Inc., USA) as described (36, 43).

### Colony Formation

HCC cells were seeded in 6-well tissue culture plates (100 cells/well) in triplicate. Colonies with more than 50 cells were counted after 2 weeks. The cells were fixed with 75% ethanol for 30 minutes and stained with 0.2% crystal violet (Beyotime Biotech, China) for 20 minutes (36, 43).

### Transwell Invasion Assay

A Transwell apparatus was used with 8-μm polyethylene terephthalate membrane filters (Corning Inc., USA). The upper chambers were seeded with 200 µl of serum-free medium containing serum-starved cells (HepG2 and SK-Hep-1: 1 × 10^4^ cells; Huh7: 2 × 10^4^ cells). The lower chambers were filled with 500 µl of 10% FBS-DMEM. After 24 hours, cells that invaded the lower chamber were fixed and stained with 0.2% crystal violet (Beyotime Biotech) as previously described (36). The invaded cell number from experiments in triplicate was counted in five randomly selected fields per chamber under an inverted microscope (Leica, Germany).

### RNA Isolation and RT–qPCR

Cells were harvested for RNA isolation using an RNeasy Mini Kit (QIAGEN, USA), and first strand cDNA was synthesized with the Superscript First-Strand Synthesis System (Invitrogen). RT–qPCR was performed using primers as previously described (36). The relative expression level of each mRNA was normalized to *β-actin* using the 2^-ΔΔCt^ method.

### Methylation-Specific qPCR

DNA extraction, bisulfite modification and MSP-PCR were performed as previously described (36, 43). Genomic DNA was extracted from tissues using the QIAamp DNA Mini Kit (Qiagen), followed by quantitative analysis using a NanoDrop 2000 spectrophotometer (Thermo Fisher Scientific, USA). Bisulfite modification of DNA was performed using a Zymo DNA Methylation Kit (Zymo Research, USA). The positive and negative template controls were the Human Methylated & Nonmethylated DNA Set (Zymo Research). MSP-qPCR was performed by using methylated or unmethylated primer pairs specifically for *PRSS3* (36) and *β-actin* (43). The relative level of methylation and unmethylation of *PRSS3* was normalized to *β-actin* using the 2^-ΔΔCt^ method.

### Methylated DNA Immunoprecipitation-qPCR

Genomic DNA was extracted from the HCC cells. The purified DNA was then sonicated into 200~1000 bp fragments. A 10% sonicated DNA sample was kept as an input control. The denatured DNA fragments (input fractions) were incubated with 2 μg anti-5-methylcytidine (5mC) (Active motif, USA) or 2 μg control IgG (Sigma–Aldrich) monoclonal antibodies at 4°C overnight, followed by precipitation using protein A beads. After washing, immunoprecipitated DNA (IP fractions) and the input control fraction were purified by using a QIAquick purification kit (QIAGEN) and analyzed by qPCR using the following primers: F: 5’- CTGTGATGGAGAGGGGGTTC -3’; R: 5’- GAGTAGTGTGCGCATCGGT-3’.

### Western Blotting

HCC cells were lysed in RIPA buffer (Beyotime Biotech) containing protease and phosphatase inhibitors (Sigma–Aldrich). Equal amounts of total protein were loaded on and separated by sodium dodecyl sulfate–polyacrylamide gel electrophoresis (SDS–PAGE) and then transferred onto polyvinylidene difluoride membranes using a Bio–Rad Mini PROTEAN 3 system. The membranes were blocked for 1 h in PBS containing 5% milk (v/v) and 0.1% Tween-20 (v/v) and incubated with the indicated primary antibodies against PRSS3 (Cat. ab107430, Abcam) and PRSS3 isoforms (**Figure S1C**) overnight at 4°C, followed by incubation for 1 h with the appropriate secondary antibodies. Immunoreactive bands were visualized by using the Amersham ECL Western Blotting Detection Kit according to the manufacturer’s instructions. β-Actin served as a loading control.

### Tumorigenicity

The animal handling and all *in vivo* experimental procedures were approved by the Institutional Animal Ethics Committee of Beijing Chest Hospital. Huh7 cells (2 × 10^6^) with or without constructs suspended in 0.1 ml PBS were injected into the subcutaneous flanks of 4-week-old Balb/c female athymic mice (Vital River Laboratories, Beijing, China). The tumor diameters and body weights of nude mice bearing HCC xenograft tumors were measured and documented every 3 days until the animals were sacrificed at day 15. HCC tumor xenografts were isolated and weighed. Tumor volume was calculated by measuring the longest (a) and shortest (b) diameters of the tumor and calculated by the formula: ab2/2.

### Statistical Analysis

The data are expressed as the means ± standard deviation (SD) of at least three independent experiments. *PRSS3* transcript expression, epigenetic alterations and associated clinicopathological correlations were analyzed by using the two−tailed Student’s t−test, Wilcoxon rank sum test, one−way analysis of variance (ANOVA) with Tukey’s *post-hoc* test, Spearman rank test and Fisher’s exact test, or χ^2^ or Fisher’s exact tests. Cancer-related survival was analyzed using the Kaplan–Meier method and was compared using log-rank tests. Statistical significance was considered when *P* < *0.05*. All statistical analyses were performed using SPSS version 23.0 (IBM Corp.).

## Results

### Aberrant Differential Expression of *PRSS3* in HCC Was Attributable to its Transcript Heterogeneity in Human HCC

**Figure 1:**
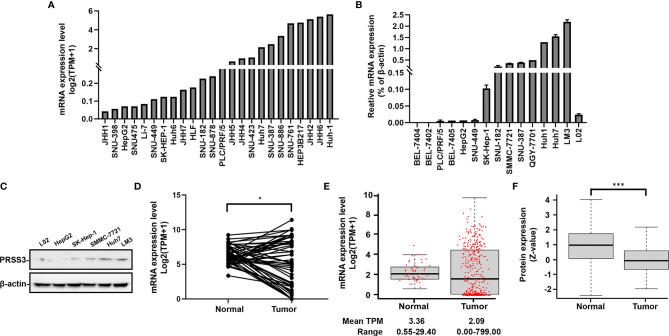
*PRSS3* expression in human HCC cell lines and tissues. **(A)** The mRNA level of *PRSS3* expression in 24 HCC cell lines using RNA-seq data extracted from the DepMap website. The expression bar chart is sorted by *PRSS3* mRNA expression levels processed on a log2 (TPM+1) scale. TPM: transcripts per million. **(B)** RT–qPCR analysis of *PRSS3* expression in 14 HCC cell lines and the human fetal liver cell line L02. The relative expression of *PRSS3* mRNA was normalized to *β-actin*, presented as the mean ± SD from three independent experiments performed in triplicate. **(C)** Western blot analysis of PRSS3 in HCC cell lines and L02 cells. **(D, E)** The RNA-seq data were sourced from FIREHOSE (**Table 1**). *PRSS3* expression in 50 pairs of tumors and matched solid normal tissues from patients with HCC **(D)** or extensively in 371 tumor samples *versus* 50 normal tissues from HCC patients **(E)**. * *P* < 0.05, by Student’s *t* test. **(F)** PRSS3 protein expression in human HCC (n = 165) in comparison with normal liver tissues (Normal) (n = 165), based on data from UALCAN portal analysis of CPTAC Confirmatory/Discovery dataset. Z-values represent the standard deviation from the median across samples for a given cancer type. Log2 spectral count ratio values from CPTAC were first normalized within each sample profile and normalized across samples. ***P < 0.001, by the Wilcoxon rank sum test.

The RNA-seq data from the Cancer Model Repository (LIMORE) and the DepMap portal revealed that *PRSS3* as a whole was differentially expressed in HCC cell lines (**Figure S1A**, **Figure 1A** and **Table S1**). RT–qPCR validation showed that the expression levels of *PRSS3* ranged from very low (*PRSS3^Low^*) to very high (*PRSS3^High^*) compared to human fetal liver L02 cells (**Figure 1B**). Western blot using an anti-PRSS3 antibody confirmed the differential expression of PRSS3 at protein level (**Figure 1C**). Comparative analysis using TCGA RNA-seq data from FIREHOSE Broad GDAC showed divergent features of *PRSS3* expression in HCC tissues compared to their matched nontumor tissues (n=50) (**Table S2** and **Figure 1D**). This was further evidenced by the analysis of more HCC tissue specimens (tumor =371) (**Table 1**, **Figure 1E**). The GEPIA portal combined TCGA with GTEx RNA-seq datasets showed that *PRSS3* expression was more varied in HCC tissues (n=369) than in normal controls (n=160) (**Figure S1B**) (38, 41). Although not statistically significant, the *PRSS3* mRNA level was lower but had an extraordinarily wide range in HCC tissues compared to normal tissues, consistent with the analysis of CPTAC data showing that PRSS3 protein was more highly expressed in normal human live tissues than in HCC tissues (**Figure 1F**). These results suggest that *PRSS3*, as a differentially expressed gene (DEG), was aberrantly and divergently expressed in HCC.

**Table 1:** Correlation between the mRNA levels of PRSS3 transcripts and clinicopathologic characteristics in patients with HCC.

Characteristics	N	*PRSS3* Expression		P
		High	Low	
		N (%)	N (%)	
**Total**	371	184 (49.6)	187 (50.4)	
**Gender**				
Male	250	123 (49.2)	127 (50.8)	
Female	121	61 (49.6)	60 (50.4)	0.9137
**Cancer stage**				
I	171	82 (47.9)	89 (52.1)	
II	86	43 (50.0)	43 (50.0)	0.8591
III	85	46 (54.1)	39 (45.9)	0.4259
IV	5	3 (60.0)	2 (40.0)	0.6736
Undefined	24	10 (41.7)	14 (48.3)	
**Tumor grade**				
I	55	21 (38.2)	34 (61.8)	
II	177	88 (49.7)	89 (50.3)	0.1794
III	122	67 (54.9)	55 (45.1)	0.0576
IV	12	5 (41.7)	7 (58.3)	1
Undefined	5	3 (60.0)	2 (40.0)	

The TCGA-LIHC data (version 28/01/2016) and clinical parameters of HCC patients were downloaded from the FIREHOSE Broad GDAC. After removing 2 samples of recurrent solid tumor tissues in the dataset, the remaining 421 samples included 50 matched pairs of primary solid normal and liver tumor tissues and 321 additional tumor specimens. The RNA level of PRSS3 expression was processed as TPM. High or low expression of PRSS3 (PRSS3^High^ or PRSS3^Low^) was classified based on the cutoff value of the median expression level of PRSS3 in the samples. The statistical significance of PRSS3^High^ or PRSS3^Low^ with clinicopathologic parameters of HCC patients was determined by χ2 tests.

**Figure 2:**
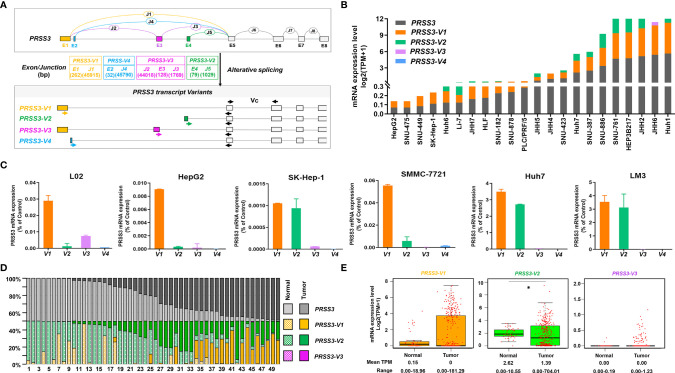
The expression of *PRSS3* splice variants in human HCC cells and tissues. **(A)** A schematic overview of the human *PRSS3* gene structure and its splicing variants (*SVs*) and the designed RT–qPCR primers. The top diagram represents the genomic organization of *PRSS3*. Alternative splicing within the 5’ region of the *PRSS3* gene leads to the creation of *PRSS3-V1 ~ -V4*. The exons and introns are represented as boxes and lines (not drawn to scale). E1-E8: Exons; J1-J8: Junctions. E5-8: gray boxes common to all four variants. E1 to 4: sequence-specific for *PRSS3-V1* (brown)*, -V4* (blue)*, -V3* (purple), and *-V2* (green), respectively. Arrowheads indicate primer set locations used for amplification of *PRSS3*-*SVs*. Forward primers were designed specifically for *PRSS3-SVs*. Reverse primers were common to all. *Vc*: RT–qPCR primer set common to *PRSS3-SVs*. **(B)** Expression level of *PRSS3-SVs* in HCC cell lines. Data from the DepMap (**Table S1**). **(C)** RT–qPCR of *PRSS3* transcripts expressed in the human fetal liver cell line L02 and HCC cell lines. The relative expression level of each mRNA was normalized against *β-actin*. **(D)** Comparison of the mRNA expression of *PRSS3* and its transcript variants in 50 paired HCC and normal liver tissues (**Table S2**). The relative percentage of *PRSS3* transcripts expressed in each paired sample (TPM scale) was visualized by a 100% stacked bar graph. **(E)** The mRNA expression of *PRSS3* transcripts in HCC tissues (n=371) and normal liver tissues (n=50) based on data from FIREHOSE. The relative transcript level is presented as a log2 (TPM+1) scale. *P < 0.05 by Wilcoxon rank sum test.

To explore the molecular basis of the divergent expression of *PRSS3* in HCC, we dissected the expression of *PRSS3-V1~-V4* in HCC (14, 15, 17–20) (**Figure 2A**). Analysis of the DepMap data revealed that in 24 HCC cell lines, *PRSS3-V2* and/or *-V1* were two major transcripts contributing to the expression of *PRSS3* because *PRSS3-V3* was infrequent and/or poorly expressed, while *PRSS3-V4* was absent (**Table S1** and **Figure 2B**). RT–qPCR showed that despite almost undetectable *PRSS3-V4* and very low expression of *PRSS3*-*V3* in all cell lines, *PRSS3-V1* was expressed at low levels in L02 cells, whereas *PRSS3-V1* and *-V2* were minimally expressed in HepG2, SK-Hep-1 and SMMC-7721 cells but highly expressed in Huh7 and LM3 cells (**Figure 2C**). Western blot using antibodies against PRSS3-V1 to -V4 showed that PRSS3-V1 and -V2 were detected in Huh7 and LM3 cells (**Figure S1C**), in parallel to their mRNA levels. Through comparative analysis of the expression levels of *PRSS3* transcripts, including *PRSS3* and its four *SVs*, in 50 paired tissue samples, we found that *PRSS3-V2* and -*V1* were predominantly present in both normal and tumor tissues (**Table S2** and **Figure 2D**). **Figure 2E** shows that the expression of *PRSS3-SVs* (no data available for *PRSS3-V4*) tended toward bipolarity in 371 HCC tissue samples compared to normal liver tissues, although only *PRSS3-V2* expression was significantly decreased. Coexpression analysis of both HCC cell lines and tissues summarized in **Table 2** further showed that the highest contributor of *PRSS3-SVs* to *PRSS3^High^* was *PRSS3-V2*, suggesting its expression dominance in *PRSS3^High^* in HCC. Moreover, *PRSS3^Low^* also resulted from decreased expression of *PRSS3-V2* and/or *-V1* because *PRSS3-V3* was minimally expressed in HCC and minimally affected the eventual expression of *PRSS3*, although *PRSS3-V3^Low^* was most frequently associated with *PRSS3^Low^*. These results thereby revealed disruption of *PRSS3* transcripts toward bipolar expression contributing to aberrant and differential expression of *PRSS3* in HCC, in which *PRSS3-V2* and/or *PRSS3-V1* were dominant transcripts leading to *PRSS3* expression.

**Table 2:** Predominance and coexpression of *PRSS3* transcripts in HCC cell lines and tissues.

	Cell lines (n =24)				Tissue specimens (n =371)			
Transcript(s)	High	%	Low	%	High	%	Low	%
***PRSS3***	12	100	12	100.00	184	100.00	187	100.00
***PRSS3-V1***	9	75.00	9	75.00	163	88.59	166	88.77
***PRSS3-V2***	10	**83.33**	9	75.00	173	**94.02**	175	93.58
***PRSS3-V3***	1	8.33	11	**91.67**	48	26.09	185	**98.93**
***PRSS3-V1+V2***	8	**66.67**	6	50.00	153	**83.15**	156	83.42
***PRSS3-V2+V3***	0	0.00	9	75.00	45	24.46	173	92.51
***PRSS3-V1+V3***	1	8.33	9	75.00	46	25.00	165	88.24
***PRSS3-V1+V2+V3***	0	0.00	6	50.00	43	23.37	155	82.89

HCC cell lines and tumor samples were classified into high or low groups in accordance with the expression of PRSS3 transcripts (median expression level as cutoff value). The details are listed in **Table 1**, **Tables S1** and **S3**.

Bold values show the highest frequency (%) of either highly or lowly expressed PRSS3-SV or coexpressed PRSS3-SVs in the HCC cell lines or tissues.

### CpG Site-Specific Methylation Regulated the Expression Divergence of *PRSS3-SVs* in HCC

**Figure 3:**
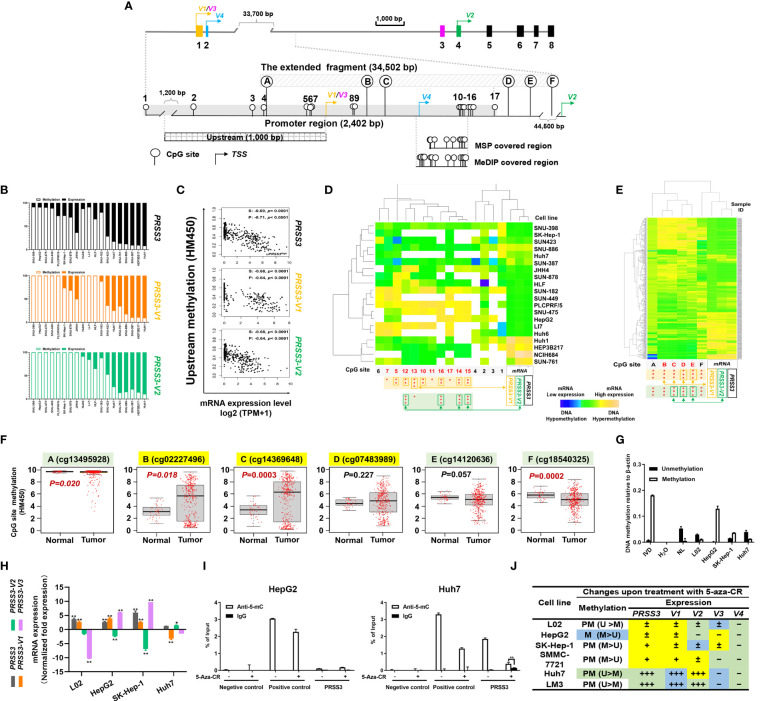
CpG methylation in the regulation of *PRSS3* transcript expression in HCC. **(A)** Schematic of the *PRSS3* 5’-genomic region including the extended promoter region and upstream and extended fragments. The promoter region (-1749 to 653 bp) shared by *PRSS3*-*V1*/*3* contains 17 CpG sites (CpGs), including 5 CpGs (CpG sites 2-7) in the 1 kb upstream fragment (-1000 bp from the TSS of *PRSS3*-*V1*/*3*). The extended fragment includes 6 CpGs (defined as **A–F**) scattering around a broad genomic region approximately 34.5 kb from -170 to 34,654 bp of the *PRSS3*-*V1*/*3* TSS but -10,643 bp from the TSS of *PRSS3*-*V2*. The genomic position of each CpG site is shown relative to the TSS of *PRSS3*-*V1*/*3* (**Table S4**). Primer-covered regions for MSP-qPCR and MeDIP-qPCR are shown. **(B)** 1 kb upstream methylation normalized as a percentage relative to *PRSS3* expression in HCC cell lines visualized by a 100% stacked bar graph. **(C)** Spearman and Pearson correlation analysis of 1 kb upstream methylation associated with *PRSS3* expression in human primary liver tumor samples (n=371). **(D, E)** Clustered heatmap of the correlation between CpG site methylation and *PRSS3* transcript expression. Data were visualized by using correlation as a distance function for heatmap cluster analysis of CpG methylation at the promoter in 20 HCC cell lines **(D)** and at the extended fragment in HCC tissue specimens (n=414) **(E)**. In the heatmap, blue indicates low, green indicates intermediate and yellow indicates high DNA methylation or mRNA values. Rows: CpG sites arranged based on the correlation between the methylation and mRNA expression levels of *PRSS3* transcripts. The values of DNA methylation levels were renormalized with mean=0 and standard deviation=1. Columns: HCC cell lines or tissue specimens. The statistical significance of correlation coefficients between CpG sites (red) and mRNA expression of *PRSS3* transcripts are shown at the bottom. *P < 0.05, **P < 0.01, ***P < 0.001 (**Figures S3, S4** and **Table S5**). **(F)** Association analysis of CpG site methylation with *PRSS3-SV* expression in 414 HCC tissue specimens compared with 41 normal controls (Wilcoxon rank sum test). **(G)** MS-qPCR of *PRSS3* methylation in HCC cell lines and L02 cells. *In vitro* methylated DNA (IVD) and normal human peripheral lymphocyte DNA (NL) served as positive and negative methylation controls, respectively. **(H)** RT–qPCR of the expression of *PRSS3* transcripts in HCC cell lines and L02 cells upon treatment with the epigenetic reagent 5-aza-CR (5 μM, 96 h). *P < 0.05, **P < 0.01 by Student’s *t* test. **(I)** MeDIP-qPCR to analyze 5-mC-enriched genomic DNA associated with the extended promoter region in HCC cell lines and L02 cells after 5-aza-CR treatment. **P < 0.01 by Student’s *t* test. **(J)** In the summary table, the differential expression changes of *PRSS3* transcripts responding to treatment with 5-aza-CR are visualized with symbols and colors. Methylation was defined as partial methylation (PM) or methylation (M) based on the MSP results. *PRSS3* expression: “–”, < 0.001%; “±”, 0.001-0.05%; “+”, > 0.05%; “+++”, > 1%. The fold changes upon 5-aza-CR treatment are shown in color: yellow, upregulation; green, downregulation; blue, no change.

We previously observed epigenetic silencing of *PRSS3* in HCC (32, 34–36). However, methylation in association with the expression of its *SVs* has never been addressed. We next assessed the contribution of DNA methylation to the expression of *PRSS3-SVs* based on the data available from DepMap and FIREHOSE (39, 40) for three genomic regions in *PRSS3*. These were referred to as the promoter region and upstream and extended fragment, respectively (**Figure 3A**). The promoter region approximately 2400 bp (-1749 to 653 bp) around the TSS shared by *PRSS3*-*V1*/*3* contains 17 CpG sites (CpGs 1-17), including 5 CpGs (CpGs 2-7) in the 1 kb upstream fragment (-1000 bp to the TSS of *PRSS3*-*V1*/*3*), while the extended fragment includes 6 CpGs (defined as A, B, C, D, E and F) scattering around a broad genomic region approximately 34.5 kb in scale from -170 to 34,654 bp of the TSS of *PRSS3*-*V1*/*3* but still -10,643 bp upstream of the TSS of *PRSS3*-*V2*. The genomic position of each CpG site is shown in **Figure 3A** relative to the TSS of *PRSS3*-*V1*/*3* (**Table S4**).

Association analysis demonstrated an inverse association between the upstream methylation and mRNA expression of *PRSS3* and its transcripts *PRSS3-V1* and -*V2* that could distinguish HCC cell lines phenotypically between hypermethylation of *PRSS3^Low^* (m*PRSS3^Low^*) and hypomethylation of *PRSS3^High^* (um*PRSS3^High^*) groups (**Figure 3B**). The pattern of m*PRSS3^Low^* versus um*PRSS3^High^* was further confirmed in tumor samples showing more similarity between *PRSS3* and *PRSS3*-*V2*, whereas *PRSS3*-*V1* was more phenotypically defined with m*PRSS3^Low^* and um*PRSS3^High^* groups (**Figure 3C**). Together with the intragenic methylation associated with *PRSS3* expression shown in our previous study (35), these results support the regulatory effect of DNA methylation on *PRSS3* transcripts.

Unsupervised clustering combined with Spearman correlation analysis of CpG site methylation and expression of *PRSS3* transcripts in HCC cell lines revealed that among 17 CpGs distributed in the promoter region, methylation occurring at CpG sites 5-17 (-89~653 bp from the TSS of *PRSS3*-*V1/V3*) (**Table S4**) was inversely correlated with the mRNA expression level of *PRSS3*-*V1*, while methylation at CpG sites 12-16 (522 to 564 bp to *PRSS3*-*V1* TSS) was highly related to *PRSS3*-*V2* expression (**Figure 3D** and **Figure S2**, **Table S5**). No associative comparison was conducted on *PRSS3*-*V3* and *-V4* due to their rare expression in HCC. This result confirms the patterns of m*PRSS3^Low^* versus um*PRSS3^High^* in HCC cells. However, only CpG site 5 in the upstream was significantly associated with the expression of *PRSS3*-*V1* (**Figure 3D**, and **Figure S2**), suggesting CpG site-specific regulation of *PRSS3* transcript expression in HCC cells. Moreover, despite a positive association shown at CpG site F, methylation at CpG sites A-E was negatively correlated with *PRSS3* expression (**Figure 3E** and **Figure S3A**), in which the associative significance of site methylation with *PRSS3* and *PRSS3*-*V2* was B, C, D, E but reversed for *PRSS3*-*V1* (**Figure S3B**). CpG site methylation at the extended fragment of *PRSS3* was decreased at site A, increased at B, C and D, and then decreased at E and F in HCC tumors compared to normal controls (**Figure 3F**). The CpG site methylation in the *PRSS3* promoter region from -89 bp (CpG site 5) to 785 bp (CpG site E) to the TSS of *PRSS3*-*V1*/*3* associated with the expression of *PRSS3* transcripts suggests an epigenetic promoter contribution to divergent expression of *PRSS3* transcripts in HCC (**Figure 3A**).

We then examined the methylation-specific effect on *PRSS3* expression using qPCR-based assays (**Figure 3A**). MSP-qPCR showed that *PRSS3* was hypermethylated in *PRSS3^Low^* cell lines (HepG2, SK-Hep-1) but hypomethylated in *PRSS3^high^* Huh7 cells compared to L02 cells (**Figure 3G**), consistent with previous observations (36). **Figure 3H** reveals that treatment with the DNA methyltransferase inhibitor 5-aza-CR caused significant upregulation of *PRSS3* expression in *PRSS3^Low^* cell lines but had no effect on *PRSS3^High^* Huh7 cells. Notably, a bipolar expression pattern was observed in *PRSS3^Low^* cell lines upon 5-aza-CR treatment, showing significant upregulation of *PRSS3-V1* and *-V3* opposite to downregulation of *PRSS3-V2*, eventually integrative to the upregulation of *PRSS3*, whereas the treatment had no effect on *PRSS3^high^* Huh7 cells, actually due to integration between upregulation of *PRSS3-V2* and downregulation of *PRSS3-V1* and -*V3*. MeDIP-qPCR further showed that the anti-5-methylcytosine (5-mC) antibody significantly enriched fewer genomic DNA fragments in HepG2 cells but not in Huh7 cells upon 5-aza-CR treatment (**Figure 3I**), suggesting that 5-aza-CR was effective in the expression of *PRSS3* specifically by altering DNA methylation in this promoter region. Although the expression of *PRSS3-V3* in L02 or *PRSS3-V2* in HepG2 and SK-Hep-1 cells was too low to take into account its decreased significance level, these results are consistent with bioinformatic analysis of HCC cell lines and tissues, as well as our previous report (36), suggesting that methylation occurring at this region is more critical for epigenetically controlling *PRSS3* transcript activities in HCC. As shown in the summarized table (**Figure 3J**), the divergence of *PRSS3* transcript expression and their response to 5-aza-CR treatment was negatively associated with site-specific CpG methylation, which eventually determined the expression level of *PRSS3* as a whole. These results suggest that differential methylation of the promoter controls the expression of *PRSS3-SVs* in a site-specific manner in HCC.

### *PRSS3-V2* Exerts Oncogenic Functions Distinct From the Tumor-Suppressive Effects of *PRSS3-V1* and *PRSS3-V3* in HCC Cells

**Figure 4:**
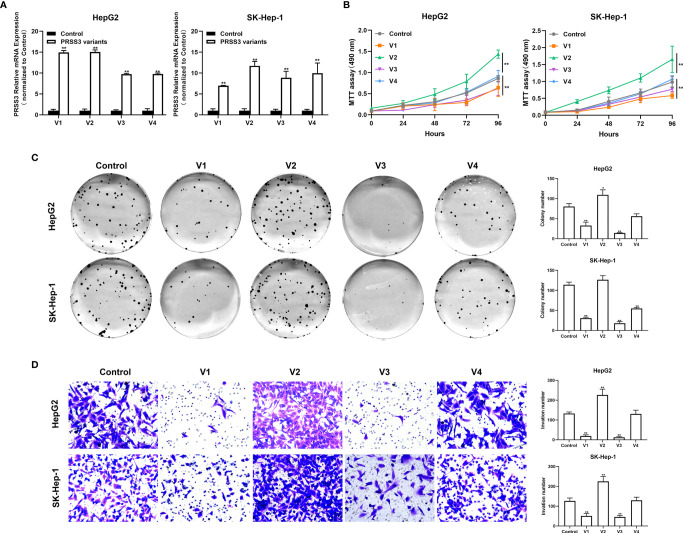
Effects of ectopically expressed *PRSS3* transcripts on HCC cell malignancy. The *PRSS3* splicing variants were separately transfected into HepG2 and SK-Hep-1 cells to establish stable cell lines with individual overexpression of either *PRSS3-V1* to *-V4* (*V1* to *V4*) or vector control (Control). **(A)** The mRNA expression levels of *PRSS3* transcripts in the transfected cells were measured by RT–qPCR and quantified relative to the control cells (Student’s *t* test). **(B)** Cell viability of HepG2 and SK-Hep-1 cells with ectopic expression of either *PRSS3* transcript was detected by MTT assays compared with the vector control (two‐tailed Student’s *t*‐test). **(C)** Colony assays showing the colony formation of HepG2 and SK-Hep-1 cells after overgrowing for 2 weeks. Representative images are presented in the left panel; quantitation of the colony numbers is shown on the right. **(D)** Transwell invasion assay assessing cell invasion capacity following transfection of *PRSS3* transcripts. Left panel: representative image at 200× magnification; right panel: quantitation of the migrated cells. One−way ANOVA with Tukey’s *post-hoc* test was calculated for the transfected cells compared with the vector control in **(C, D)**. *P < 0.05, **P < 0.01, *versus* control. Data are presented as the mean ± SD of three independent experiments performed in triplicate.

The functional role of *PRSS3-SVs* was assessed by transfecting *PRSS3-V1* to -*V4* into *PRSS3^Low^* HepG2 and SK-Hep-1 cells (defined as V1 to V4) (**Figure 4A** and **Figure S4A**). MTT assays showed that ectopic expression of *PRSS3-V1* or *PRSS3*-*V3* significantly inhibited HCC cell proliferation in contrast to notably enhancing the effect of ectopic *PRSS3-V2* expression or nonfunctional *PRSS3-V4* on HCC cell proliferation compared to the vector controls (**Figure 4B**). Moreover, the results of the clone formation assay showed that overexpression of *PRSS3-V1* or *PRSS3*-*V3* remarkably diminished the number of colonies of HCC cells compared with the control group, but *PRSS3-V2* overexpression resulted in an increased number of colonies only effectively in HepG2 cells. However, ectopic *PRSS3-V4* significantly reduced clone formation in SK-Hep-1 cells but had no effect in HepG2 cells (**Figure 4C**). Transwell assays further showed an inhibitory effect of *PRSS3-V1* or *PRSS3*-*V3* on HCC cell migration, in contrast to *PRSS3-V2*, which showed an enhanced effect in the cells (**Figure 4D**). These results suggest a tumor-suppressive effect of *PRSS3-V1*/*V3* versus an oncogenic effect of *PRSS3-V2* in HCC cells.

**Figure 5:**
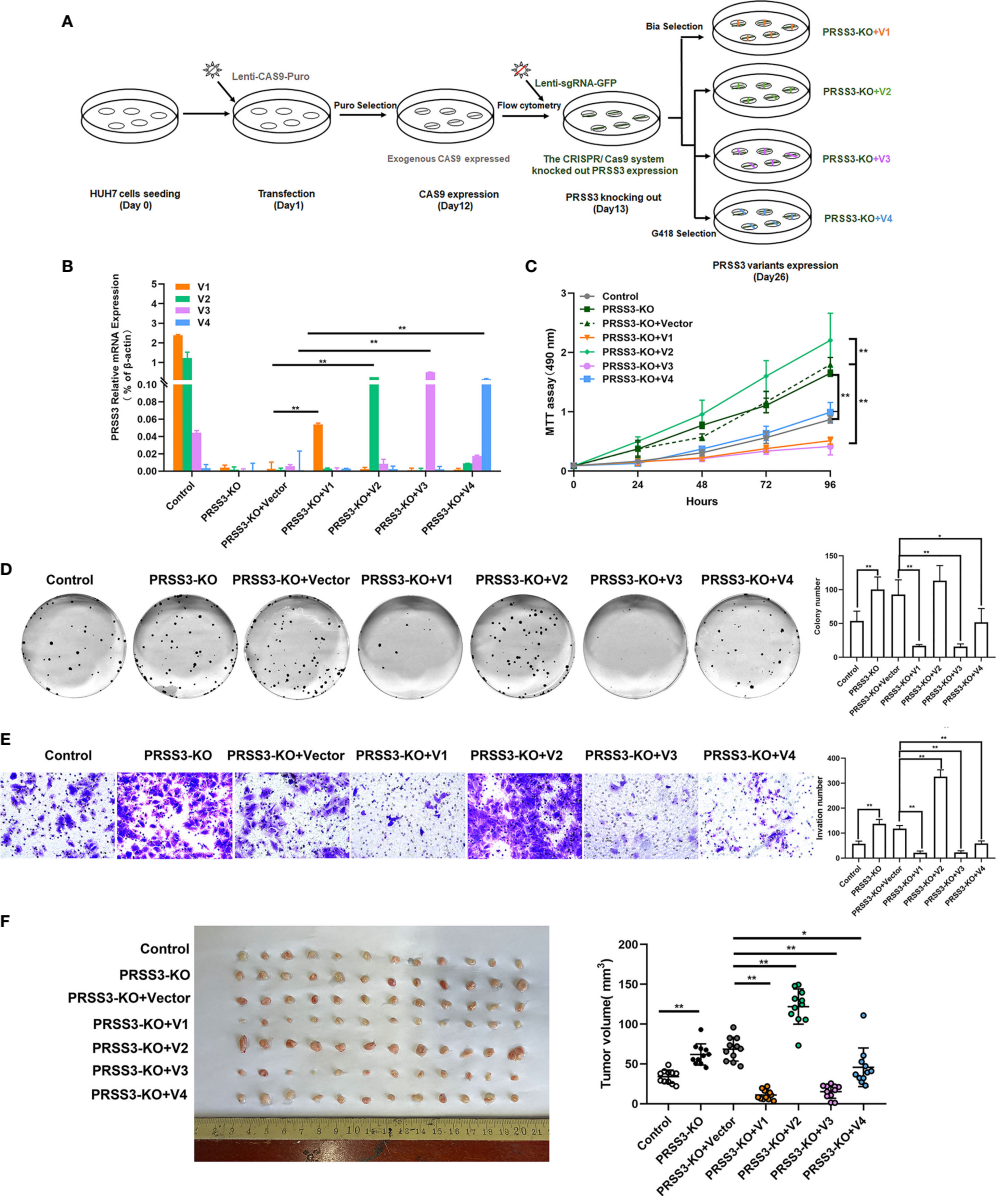
Functional divergence of *PRSS3* transcript variants in a gain- and loss-of-function cell model. **(A)** Schematic of the workflow for the construction of a cell model by endogenous deletion and then ectopic expression of the *PRSS3* transcript in Huh7 cells (*PRSS3 ^KO+V^* cell model). Genomic deletion of *PRSS3* transcripts by targeting the common exon 5-8 region in *PRSS3^High^* Huh7 cells was performed using the CRISPR/Cas9 system, followed by transfection with *PRSS3-V1* to *PRSS3-V4* constructs (*PRSS3 ^KO+V1^* to *PRSS3^KO+V4^*) or vector control (*PRSS3^KO+C^*). Puromycin (Puro), blasticidin (Bla) and geneticin (G418) were used for selection of the transduced cells. **(B)** RT–qPCR analysis of *PRSS3* mRNA expression in the transfected cells. The relative mRNA expression of *PRSS3* transcripts normalized to *β-actin* (Student’s *t* test). **(C)** MTT assays showed the viability of Huh7 cells (two‐tailed Student’s *t*‐test). **(D)** Colony formation of Huh7 cells for 2 weeks. Left panel: representative image; Right panel: The colony numbers counted. **(E)** Transwell invasion assay assessing the invasion capacity of Huh7 cells upon transfection. Left panel: representative images at 200× magnification; right panel: quantitation of the invaded cells. One−way ANOVA with Tukey’s *post-hoc* test was calculated for the transfected cells compared with the vector control in **(C–F)**. *P < 0.05, **P < 0.01, versus control. Data are presented as the mean ± SD of three independent experiments performed in triplicate. **(F)** Effects of *PRSS-SVs* on HCC tumorigenicity using the *PRSS3 ^KO+V^* Huh7 cell model. Photographs (left panel) and tumor volumes (right panel) of dissected xenograft tumors from different groups of nude mice (n=12) after sacrifice. *P < 0.05, **P < 0.01, one−way ANOVA with Tukey’s *post hoc* test.

To further define the phenotypic properties of *PRSS3-SVs* in HCC cells, we established a *PRSS3^KO+V^* cell model in which each *PRSS3* transcript construct was separately transfected after endogenous *PRSS3* was deleted through the CRISPR/Cas9 system (**Figure 5A**). RT–qPCR showed that all the detected *PRSS3* transcripts were effectively deleted, and their constructs were stably expressed in Huh7 cells, designated *PRSS3^KO+V1^* to *PRSS3^KO+V4^*, or the vector control (*PRSS3^KO+V^*) (**Figure 5B**), which was further confirmed by Western blot analysis of PRSS3 protein isoforms (**Figure S4B**). Functional assays, as shown in **Figure 5C–E**, revealed that *PRSS3* deletion in Huh7 cells facilitated cell proliferation, colony formation and migration, which were abolished by re-expression of *PRSS3-V1* or *PRSS3*-*V3*. Ectopic re-expression of *PRSS3-V2* augmented the *PRSS3-*deletion effects on cell proliferation and, remarkably, on the migration of *PRSS3^KO^* Huh7 cells. Unexpectedly, *PRSS3-V4* re-expression did not affect Huh7 cell proliferation but resulted in significant inhibition of *PRSS3^KO^* Huh7 cell activity. To analyze the functional roles of the *PRSS3* variants in tumor growth *in vivo*, a tumor xenograft assay was performed by injecting *PRSS3^KO+V^* cells into nude mice (**Figure S5**). Consistent with *in vitro* findings, *PRSS3* deletion favored xenograft tumor growth formed by Huh7 cells, with a significant augmentation by re-expression of *PRSS3-V2* (*PRSS3^KO+V2^*), whereas re-expressing either *PRSS3-V1* or *PRSS3*-*V3* (*PRSS3^KO+V1/3^*) in the cells caused a marked inhibitory effect on xenograft tumor growth in contrast to a minimal role of *PRSS3^KO+V4^* (**Figure 5F**). These results exclusively demonstrate the dual roles of *PRSS-SVs* in HCC cells, and divergent disruption of *PRSS3* transcripts may be integrated to establish their functional heterogeneity in HCC cells.

To explore the possible mechanisms by which the *PRSS3* transcript variants exerted the dual effects on hepatocarcinogenesis, potential *PRSS3*-targeted downstream genes were sorted using network analysis of TCGA-LIHC tissue dataset available from SEEK (http://seek.princeton.edu) (**Figure S6A**), among which 8 key hub genes were shown in most association with *PRSS3* transcripts (except *PRSS3-V4*) either positively (*F2RL1, SMPDL3B, DUOX2, SLC43A3, TMEM45A* and *VNN1*) or negatively (*GLUL* and *NKD1*) in the network (**Figure S6B**), consistent with the validation in HCC cells using the CCLE dataset (**Tables S1, S7** and **S8**). In addition to a heatmap visualizing the differential expression of the hub genes (**Figure S6C**), **Figure S6D** shows significant upregulation of *F2RL1, SMPDL3B, DUOX2, SLC43A3, GLUL* and *NKD1*, but upregulation of *TMEM45A* and *VNN1* in HCC tissues compared with normal human live tissues. As shown in the summarized table based on the available data from UALCAN (https://www.ualcan.path.uab.edu/), there was a divergent association of the clinical significance between *PRSS3* and the hub genes (**Figure S6E**). For instance, the pattern of *PRSS3* downregulation associated with the clinical relevance of HCC patients was similar to that of *TMEM45A* and *VNN1*, which are positively co-expressed genes of *PRSS3* in HCC patients showing oncogenic effects on cancer-associated events (44, 45). However, *GLUL* and *NKD1*, completely opposite to *PRSS3*, showed increased expression related to clinical relevance, displaying the ability to regulate the invasion and migration of hepatocarcinoma cells (46–49). Importantly, PRSS3/MTG linked to F2RL1 (also known as PAR2), was reported to modulate inflammation and tumorigenesis in several cancer types, such as colon cancer and breast cancer (23, 24). In support of this point, Kaplan–Meier survival analysis showed divergent survival curves for HCC patients with high or low expression of the hub genes (**Figure S6F**). Kyoto Encyclopedia of Genes and Genomes (KEGG) pathway enrichment analysis indicated that these cancer-associated genes may be involved in the cell cycle and senescence through the *PRSS3-V1*-associated p53 signaling pathway, or *via* the PI3K-Akt pathway in association with *PRSS3-V2*/*PRSS3*, due to their corresponding pathways with more parallel lines (**Figure S6G**). These data therefore suggest that *PRSS3* transcripts are bifunctional, possibly *via* interplay with different cancer-associated gene pathways.

### Epigenetic Alteration of *PRSS3-V2* Is Associated With Clinical Relevance in Patients With Early HCC

**Figure 6:**
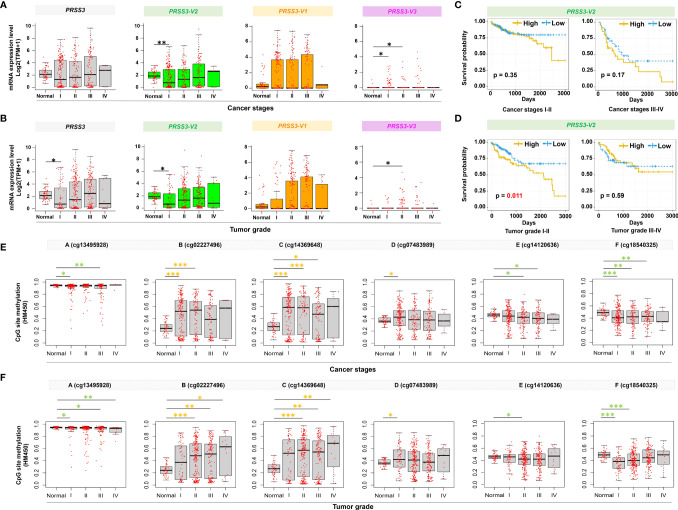
Clinical relevance of epigenetic alteration of *PRSS3-SVs* in HCC patients. **(A, B)** Box-and-whisker plot with overlay of individual data points showing mRNA expression of *PRSS3* transcripts in HCC tissues (Tumor=371) and normal controls (Normal=50), based on **(A)** cancer stages (171 stage I, 86 stage II, 85 stage IIII, 5 stage IV) and **(B)** tumor grades (55 grade I, 177 grade II, 122 grade III, 12 grade IV) (**Table 1**). **(C, D)** HCC patients were grouped into *PRSS3-V2^High^* and *PRSS3-V2^Low^* groups based on the mean value of each transcript in tumors (**Table 1**). The Kaplan–Meier method was used to determine patient survival, and the log-rank (Mantel–Cox) test was used to compare survival rates. The results of HCC patient survival curves from left to right panels: cancer stages I-II and III-IV **(C)**, tumor grades I-II and tumor grades III-IV **(D)**. **(E, F)** The association of methylation of CpG sites **(A–F)** within the extended fragment with different clinical stages **(E)** and pathological grades **(F)** in HCC tissue specimens (n=414) in comparison with normal liver control tissues (Normal=41). The data were extracted from the FIREHOSE. Statistical significance was determined by the Wilcoxon rank sum test. Asterisks in green and yellow indicate the changes in hypermethylation and hypomethylation, respectively. *P < 0.05, **P < 0.01, ***P < 0.001.

To further explore the contribution of *PRSS3* transcripts to tumor heterogeneity, we used the TCGA dataset to analyze their clinical relevance. We found that the expression of *PRSS3* and *PRSS3-V2* was similarly downregulated but with a gradually increasing tendency in HCC tumors compared with control tissues, following the progression of tumor stages (**Figure 6A**) and pathological grades (**Figure 6B**), in which *PRSS3-V2^Low^* was significantly detected in tumors of early HCC patients in contrast to *PRSS3-V2^High^* in advanced tumors. Kaplan–Meier (K-M) analysis revealed that *PRSS3-V2^Low^* was a favorable factor for the overall survival of HCC patients based on cancer stage (**Figure 6C**) and grade (**Figure 6D**), in which *PRSS3-V2^Low^* patient groups with low-grade tumors showed significantly favorable outcomes (*P*=0.011). Moreover, divergent disruption of CpG site methylation (A to F) was shown throughout the clinical progression of tumors but occurred more frequently and significantly in tumors of HCC patients with early-stage (**Figure 6E**) and lower-grade tumors (**Figure 6F**). In such tumors, alteration in CpG methylation at site D was most inversely correlated with the expression of *PRSS3* and *PRSS-V2*. Since the region located at site D was shown to be an important regulatory region specifically for epigenetic regulation of *PRSS3* transcripts (**Figure 3**), the data suggest that site-specific epigenetic alteration of *PRSS3-V2* in HCC tissues was distinct between mPRSS*3-V2^Low^* in early HCC and um*PRSS3^High^* in advanced HCC patients, in which early HCC patients with *PRSS3-V2^Low^* tumors had better outcomes.

## Discussion

Paradoxical effects of many genes have been observed during tumorigenesis (13, 50, 51). The protease PRSS3 is the first to link the enzyme to prostate cancer, leading to the development of a compound to stop PRSS3 from promoting metastasis (13, 52). Since the high similarity in both sequences and structures to different trypsinogen isoenzymes made it difficult to delineate their functionally associated transcripts distributed in different tissues (13, 16), the protumor (21–31) or antitumor properties of PRSS3 (32–36) were too sophisticated to be deciphered. In this study, we found differentially expressed *PRSS3* in HCC due to CpG methylation-mediated epigenetic dysregulation of its splice variants. Different *PRSS3-SVs* were expressed in HCC, showing a dual role in hepatocarcinogenesis that may increase phenotypic diversity. Our study uncovered epigenetic-mediated *PRSS3* transcript variance contributing to the nongenetic phenotypic diversity of HCC (50). To the best of our knowledge, this is the first study of functional dissection of the expression of *PRSS3-SVs* in cancer and thus has important implications in HCC patient-tailored management.

PRSS3 is a digestive protease with restricted expression in the pancreas. However, the preferential expression of *PRSS3-SVs* differs in human tissues, suggesting tissue-selective expression. For instance, *PRSS3-V2* was exclusively expressed in human pancreatic tissue and fluid encoding MTG (15, 53). Canonical *PRSS3-V1* was originally identified in the human brain (17, 53). *PRSS3-V3* shares the same TSS with *PRSS3-V1* but has a different in-frame exon with a deduced 261-amino acid sequence (formerly named isoform B) (19). *PRSS3-V4* was cloned from keratinocytes and shown to participate in keratinocyte terminal differentiation (20). Our study showed the differential expression of *PRSS3* as a DEG in HCC across a large expression range that could be used to phenotypically distinguish between *PRSS3^Low^* and *PRSS3^High^* HCC cells and tissues. Accordingly, we found divergent expression of *PRSS3-SVs* toward bipolarity following clinical progression from downregulation in early HCC to upregulation in advanced cancer, unveiling the molecular basis of *PRSS3* in tissue-selective expression of its splice transcripts in HCC. Despite the infrequent and/or minimal expression of *PRSS3*-*V3* and unexpressed *PRSS3-V4*, the divergent expression changes of *PRSS3-V2* and/or -*V1* were major contributors to the transcript heterogeneity of *PRSS3* in HCC. Notably, the expression of *PRSS3-SVs* was dynamically altered following clinical progression from downregulation in early HCC to upregulation in advanced cancer. *PRSS3* transcript heterogeneity was further evidenced by its divergent responses to 5-aza-CR treatment of HCC cells, distinguishing between upregulation of *PRSS3-V1* or *-V3* but downregulation of *PRSS3-V2* in *PRSS3^Low^* HCC and downregulation of *PRSS3-V1* but upregulation of *PRSS3-V2* in *PRSS3^High^* HCC. The divergent expression of *PRSS3* transcripts and their response to 5-aza-CR prompted our consideration of the effects of nongenetic heterogeneity on the chemotherapy response because this well-known anticancer drug has broad clinical applications and mis-splicing regulation, as a nongenetic mechanism is frequently linked to therapy escape (54–56). For precise evaluation of the clinical effectiveness and drug resistance by using a DEG, its functional splice variants, rather than its overall expression, need to be considered. Nevertheless, it was clear that differentially expressed *PRSS3* decreased as a whole, which was mainly attributable to its aberrant transcript variance expressed in HCC.

*PRSS3* translocates from chromosome 7q34, the loci of *PRSS1* and *PRSS2*, to chromosome 9p11.2, a region frequently containing alterations (13, 57). However, frequent genetic variations occurring in *PRSS3* have not yet demonstrated disease-associated *PRSS3* variants (https://www.nextprot.org/entry/NX_P35030/medical). Alternative splicing forms a dynamic interactome offering precise therapeutic approaches to correcting cancer-specific defects caused by mis-splicing regulation, in which epigenetics plays an essential role (9, 11, 12, 55, 58–60). Our previous study showed epigenetic silencing of *PRSS3* in HCC (36), and we reasoned that epigenetic regulation of *PRSS3-SVs* contributes to nongenetic heterogeneity in HCC. The different TSSs and start codes in *PRSS3* suggest that *PRSS3*, like the majority of protein-coding genes, tends to be regulated by multiple or alternative promoters, the usage of which provides pretranscriptional control of gene activity to express its different isoforms in a tissue-specific manner (1, 6, 9, 24). Here, we found an extended promoter region covering the upstream and intragenic regions of *PRSS3-V1*/*V3* and *PRSS3-V4*, providing a site-specific way to regulate the expression of *PRSS3-SVs*. Both HCC cells and tissues were phenotypically classified as m*PRSS3^Low^* and um*PRSS3^High^* based on CpG methylation in association with the expression of *PRSS3* transcripts. Compared to consistent upstream hypermethylation, site-specific CpG methylation in the intragenic region was found to be more associated with the expression of *PRSS3-V1* and *PRSS3-V2*, suggesting that this extended promoter region played a central role in the regulation of both *PRSS3-V1* and *PRSS3-V2*. Given that epigenetic promoter alterations can change the chromatin accessibility of transcription regulatory elements binding to transcription factors (11, 12, 50, 60–63), the upstream hypermethylation of *PRSS3* may impact tissue-specific *cis*-regulatory modules that may alter the transcriptional activity of *PRSS3-SVs* in HCC. Dynamic disruption of methylation of different CpG sites within the extended promoter region may affect the occupancy of certain transcriptional regulators or splicing factors, resulting in an alteration in exon skipping to control the expression of *PRSS3-V1* or -*V3*. Meanwhile, site-specific epigenetic control of *PRSS3-V2* suggests that the extended promoter may be a distal regulatory region in the regulation of *PRSS3-V2* through a very different epigenetic pathway (64). Consistent with this, epigenetic silencing of *PRSS3* was found in several cancer types (32–35), and our previous study showed intragenic DNA methylation within the extended promoter region contributing to *PRSS3/TRY-4* downregulation in HCC (36). This study was the first to dissect epigenetic heterogeneity in the regulation of *PRSS3-SVs*, which may provide important implications for understanding epigenetic contributions to the genomic occupancy of transcription factors during transcription, in which many events may appear to be cospliced with distant events (58, 61–63).

Many transcript isoforms can exist per gene (9–11), most of which are thought not to be functionally relevant (65). Recently, comprehensive gain- and loss-of-function studies have shown the functional importance of *SVs* in tumor heterogeneity by linking genetic variants to individual phenotypes (58–60, 66, 67). *PRSS3* appears to be transcribed differentially to display heterogeneous functions in cancer, in which a dual role or contradictory effects reported might be due to MTG (PRSS3-V2) being functionally regarded as PRSS3 (13, 16, 22, 23, 29). We hereby deciphered *in vitro* and *in vivo* functional differences among the PRSS3 isoforms by using a constructed Huh7 cell model. Despite PRSS3-V2/MTG-mediated oncogenic effects in HCC in line with the promalignant activities of MTG shown in other cancer types (13, 16, 22, 23, 29), PRSS3-V1/TRY-4 or -V3 were found to be tumor suppressors in HCC cells, while ectopic PRSS3-V4 showed an inhibitory effect on *PRSS3^ko^* Huh7 cells. *PRSS3^ko^* resulted in protumor effects in Huh7 cells, suggesting a tumor-suppressive role of PRSS3 in HCC that was attributed to the coexpression of PRSS3-V1 and PRSS3-V2, the two isoforms with opposite functionality. This is in line with our previous observations on *PRSS3/TRY-4* (36) and may explain some but not all cases of a similar phenotype with well-differentiated and/or low metastatic potential appearing in either *PRSS3^Low^* (e.g., HepG2 and SK-Hep1 cells) or *PRSS3^High^* (Huh7 cells) live cancer cell lines or a dual role of *PRSS3* contradictorily shown in carcinogenesis. To support this, corresponding clinicopathological analysis of HCC specimens compared to the normal tissue controls revealed that *PRSS3-V1* and -*V2* were main functional components of clinical relevance since *PRSS3-V1* and -*V2* were bipolarly present in either *PRSS3^Low^* or *PRSS3^High^* tissues; therefore, their abnormal coexpression could result in functional heterogeneity including insignificant or paradoxical clinical associations. However, a signature pattern of epigenetic regulation of *PRSS3* expression by site-specific CpG methylation was dynamically shown from m*PRSS3^Low^* to um*PRSS3^High^* through clinical progression, better matched to *PRSS3-V2*, suggesting PRSS3-V2 to be a more prevalent isoform functionally through clinical progression of HCC. Accordingly, significant epigenetic downregulation of *PRSS3-V2* was observed in early HCC with favorable patient outcomes. This finding supports an oncogenic role of PRSS3-V2/MTG predominantly in HCC, thus providing early diagnostic and prognostic value for HCC (16, 22, 23, 29). Thus, our study provides additional evidence supporting the hypothesis of functional hepato-heterogeneity attributed to genetic and epigenetic factors (1, 2, 4, 6).

Aberrant expression of *SVs* in cancer generates functional tumor heterogeneity resulting in cellular phenotype(s) or influencing cell fate determination (1, 4, 7, 8). In this regard, delineation of the heterogeneity of *PRSS3* expression and epigenetic regulation is critical for clarifying the molecular basis of *PRSS3* transcripts, thus facilitating functional interpretation of the paradoxical effects of *PRSS3* in cancer development. Functional classification and experimental dissection of *PRSS3-SVs* and their response to 5-aza-CR treatment distinct between *PRSS3^Low^* and *PRSS3^High^* HCC cells (such as Huh7 *versus* HepG2 cells) may be used as an experimental model for studying *PRSS3* splicing-mediated functional heterogeneity during hepatocarcinogenesis. In contrast to permanent genetic mutations, epigenetic disruptions frequently occur in early clinical stages and play an important role in modulating cell malignancy in a progressive and reversible manner. Therefore, delineation of the precise molecular mechanisms underlying epigenetic regulation of *PRSS3-SVs* could contribute to the molecular phenotypes of HCC.

This study on bioinformatic analysis of RNA sequencing data of *PRSS3-SVs* and their clinical relevance gave many insignificantly divergent results. For instance, *PRSS3^Low^* was shown in 50 paired HCC tissues, consistent with our previous observation (36) and the analyses shown in the TCGA and UALCAN portals (38). However, its decrease was no longer statistically significant in more HCC tissue specimens due to different statistical methods or integration of the RNA-seq data with different median cutoff values for extensively divergent expression of *PRSS3-SVs* in HCC specimens. Therefore, conventional parameters, such as the median cutoff values, may need to be reevaluated for grouping a DEG with divergent expression levels. Moreover, functional heterogeneity could be caused by the microenvironment enhancing the coexpression diversity of *PRSS3-SVs*. As a result, further studies with larger sample sizes of paired HCC specimens are warranted to validate our observations. Moreover, due to the lack of commercial antibodies capable of discriminating well among PRSS3 isoforms, the functional pathways corresponding to PRSS3 isoforms could not be precisely distinguished from each other. This may yield inconsistent reports of *PRSS3* effects on carcinogenesis, resulting in inconclusive informatics analyses of the molecular mechanisms related to PRSS3 isoforms. Therefore, customized generation of more isoform-specific antibodies will be the subject of our future investigation to explore the molecular mechanisms underlying the dual role of *PRSS3* transcript isoforms in cancer development.

## Conclusions

**Figure 7:**
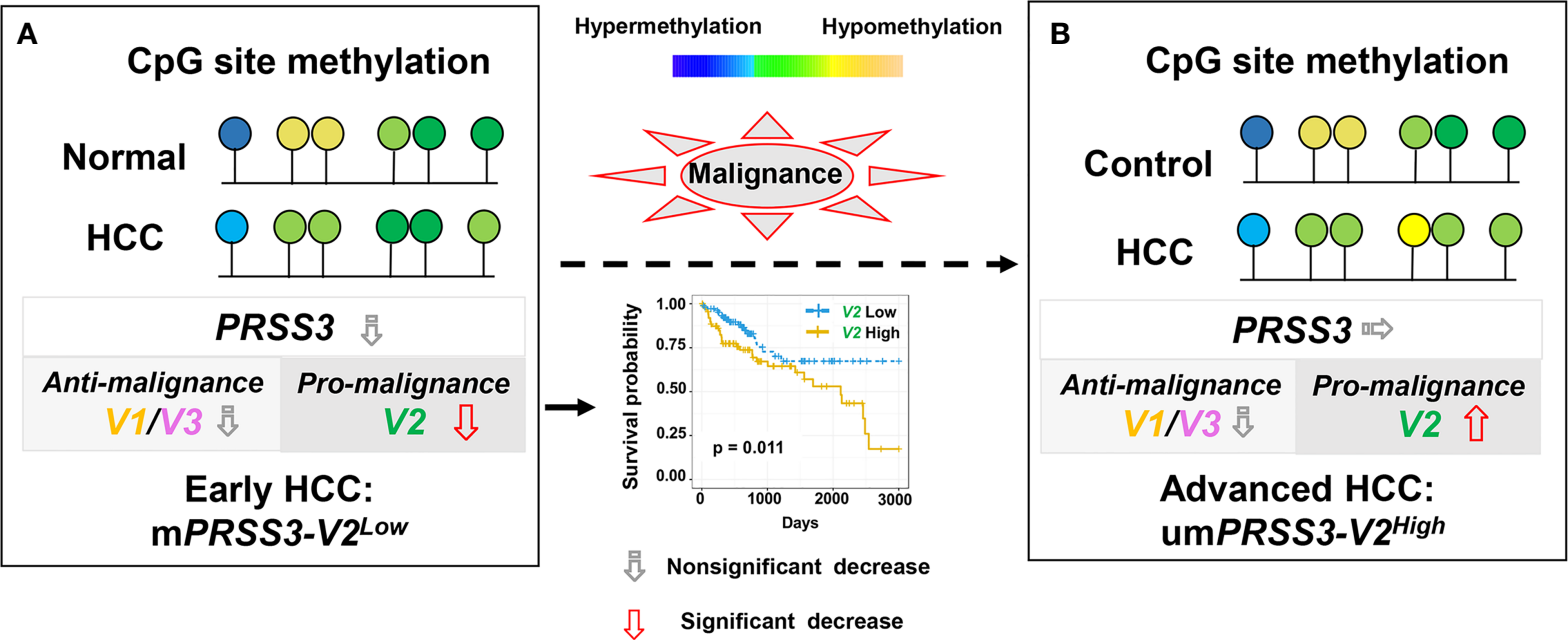
A schematic model for epigenetic dysregulation of *PPRSS3* transcripts functionally contributing to hepatocarcinogenesis and its biomarker potential. **(A)** Epigenetic silencing of *PRSS3-SVs* by site-specific CpG methylation in the tumors of patients with early HCC, in which m*PRSS3^Low^* was a potential biomarker favorable for patient survival. **(B)** Epigenetic disruption resulted in um*PRSS3^High^* in tumors of advanced HCC patients.

In summary, *PRSS3* was aberrantly expressed in HCC due to epigenetic dysregulation that was integrated with divergent expression of *PRSS3-SVs* by site-specific CpG methylation. The effects of oncogenic *PRSS3-V2* and tumor-suppressive *PRSS3-V1* in HCC cells may increase the molecular diversity and functional plasticity of hepatocarcinogenesis. Epigenetic dysregulation of *PRSS3-V2* distinct between *mPRSS3-V2^Low^* in early clinical stages and um*PRSS3^High^* in advanced tumors has potential diagnostic value for patients with early HCC (**Figure 7**).

## Data Availability Statement

The datasets presented in this study can be found in online repositories. The names of the repository/repositories and accession number(s) can be found in the article/**Supplementary Material**.

## Ethics Statement

The animal handling protocols and all *in vivo* experimental procedures were approved by the Institutional Animal Ethics Committee of the Beijing Chest Hospital.

## Author Contributions

Conceptualization, SL, HX and JH; Formal analysis, SL, HX, MP, XG, XCZ, LZ, FJ, YH, WW, JR and JH; Funding acquisition, JW, MG and JH; Investigation, SL, HX, XMZ, MP, LZ, XG, YP and ZY; Methodology, SL, XMZ, HX, MP, XW, BL, RT, XCZ, ZY and JH; Resources, KC and WG; Supervision, MG and JH; writing-original draft, SL and JH; writing-review & editing, JW and JH. All authors have reviewed and agreed to the final version of the manuscript.

## Funding

This study was funded by the Scientific Research Project of Beijing Educational Committee (Grant No. KM202110025004), the Intramural Research Funding Program from Beijing Tuberculosis and Thoracic Tumor Research Institute/Beijing Chest Hospital, National Key Research and Development Program of China (2018YFA0208902, 2020YFC2002705); Beijing Natural Science Foundation of China (7214242, 7171008), National Science Foundation of China (NSFC Grant No. 81872021, U1604281, 81672138); National Key Scientific Instrument Special Program of China (Grant No. 2011YQ03013405). KC, JH and JMW were also funded in part by Federal funds from the National Cancer Institute, National Institutes of Health, under Contract No. HHSN261200800001E and were supported in part by the Intramural Research Program of the NCI, CCR, LCIM, NIH.

## Conflict of Interest

Author WG is employed by Basic Research Program, Leidos Biomedical Research, Inc.

The remaining authors declare that the research was conducted in the absence of any commercial or financial relationships that could be construed as a potential conflict of interest.

## Publisher’s Note

All claims expressed in this article are solely those of the authors and do not necessarily represent those of their affiliated organizations, or those of the publisher, the editors and the reviewers. Any product that may be evaluated in this article, or claim that may be made by its manufacturer, is not guaranteed or endorsed by the publisher.

## Abbreviations

5-aza-CR, 5 aza-2’-deoxycytidine; CCLE, Cancer Cell Line Encyclopedia; DEG, Differentially expressed gene; DepMap, The Cancer Dependency Map; FPKM, Fragments Per Kilobase Million; GTEx, Genotype-Tissue Expression; GEPIA, Gene Expression Profiling Interactive Analysis; HCC, Hepatocellular carcinoma; LIHC, Liver hepatocellular carcinoma; MeDIP-qPCR, Methylated DNA immunoprecipitation-qPCR; mPRSS3^Low^ lowly expressed *PRSS3-SVs* with hypermethylation; MS-qPCR, Methylation-specific qPCR; MTG, Trypsin-3 isoform 2/mesotrypsinogen; *PRSS3*, Serine protease 3 gene; PRSS3, Serine protease 3; *PRSS3^High^* or *PRSS3^Low^*, Highly or minimally expressed *PRSS3*; *PRSS3-V1~-V4*, *PRSS3* transcript variants 1, -2, -3 and -4; *PRSS3-SVs*, Splice transcripts of *PRSS3;* TCGA, The Cancer Genome Atlas; TPM, Transcripts Per Million; TRY4, Trypsin-3 isoform 1/trypsinogen 4; um*PRSS3^High^*, highly expressed *PRSS3-SVs* with hypomethylation.
