# Supplementary material for: CpG Site-Specific Methylation-Modulated Divergent Expression of PRSS3 Transcript Variants Facilitates Nongenetic Intratumor Heterogeneity in Human Hepatocellular Carcinoma
Source: Front Oncol. 2022 Apr 11;12:831268. doi: 10.3389/fonc.2022.831268 (PMC9035874; doi:10.3389/fonc.2022.831268)
Supplement: Supplementary file 1 [file DataSheet_1.docx]

Supplementary Material

# Supplementary Tables

# Table S1 Expression of *PRSS3* and its transcript variants in human HCC cell lines

| Gene_ID |  | | ENSG00000010438.12 | | | | | | | | | | | | | | | | | | | | | | | | | | |  |
| --- | --- | --- | --- | --- | --- | --- | --- | --- | --- | --- | --- | --- | --- | --- | --- | --- | --- | --- | --- | --- | --- | --- | --- | --- | --- | --- | --- | --- | --- | --- |
| *Ensemble* Name | PRSS3 | | PRSS3-202 | | | PRSS3-  203 | | | | PRSS3-  201 | | | PRSS3-  204 | | | PRSS3-  205 | | | PRSS3-206 | | | PRSS3-  207 | | | | PRSS3-  208 | | | |  |
| Transcript ID |  | | ENST00000361005.5 | | | | ENST00000379405.3 | | | ENST00000342836.4 | | | ENST00000429677.3 | | | ENST00000457896.1 | | | ENST00000468152.2 | | | | ENST00000477653.2 | | | | ENST00000495682.1 | | |  |
| Refseq ID |  | | NM_007343.3 | | | | NM_002771.3 | | | NM_001197097.2 | | | NM_001197098.1 | | | PRSS3-205/002 | | |  | | | |  | | | |  | | |  |
| Transcript variant |  | | *PRSS3-V1* | | | | *PRSS3-V2* | | | *PRSS3-V3* | | | *PRSS3-V4* | | |  | | |  | | | |  | | | |  | | |  |
| Isoform |  | | PRSS3-V1 | | | | PRSS3-V2 | | | PRSS3-V3 | | | PRSS3-V4 | | | 177aa | | | no protein | | | | no protein | | | | no protein | | |  |
| JHH1 | | 0.043 | | | 0.030 | | | | 0.000 | | | 0.000 | | | 0.000 | | | 0.000 | | | 0.000 | | | | 0.000 | | | | 0.000 | |
| SNU398 | | 0.057 | | | 0.000 | | | | 0.000 | | | 0.000 | | | 0.000 | | | 0.000 | | | 0.000 | | | | 0.000 | | | | 0.060 | |
| HepG2 | | 0.070 | | | 0.000 | | | | 0.000 | | | 0.000 | | | 0.000 | | | 0.070 | | | 0.000 | | | | 0.000 | | | | 0.000 | |
| SNU475 | | 0.070 | | | 0.000 | | | | 0.000 | | | 0.000 | | | 0.000 | | | 0.140 | | | 0.000 | | | | 0.000 | | | | 0.000 | |
| LI7 | | 0.084 | | | 0.000 | | | | 0.160 | | | 0.000 | | | 0.000 | | | 0.000 | | | 0.000 | | | | 0.000 | | | | 0.000 | |
| SNU449 | | 0.111 | | | 0.000 | | | | 0.000 | | | 0.000 | | | 0.000 | | | 0.110 | | | 0.000 | | | | 0.000 | | | | 0.000 | |
| SK-Hep-1 | | 0.124 | | | 0.120 | | | | 0.000 | | | 0.000 | | | 0.000 | | | 0.000 | | | 0.000 | | | | 0.000 | | | | 0.000 | |
| Huh6 | | 0.124 | | | 0.000 | | | | 0.080 | | | 0.000 | | | 0.000 | | | 0.000 | | | 0.000 | | | | 0.000 | | | | 0.000 | |
| JHH7 | | 0.163 | | | 0.000 | | | | 0.210 | | | 0.000 | | | 0.000 | | | 0.000 | | | 0.000 | | | | 0.000 | | | | 0.000 | |
| HLF | | 0.176 | | | 0.000 | | | | 0.180 | | | 0.000 | | | 0.000 | | | 0.000 | | | 0.000 | | | | 0.000 | | | | 0.000 | |
| SNU182 | | 0.227 | | | 0.120 | | | | 0.120 | | | 0.000 | | | 0.000 | | | 0.000 | | | 0.000 | | | | 0.000 | | | | 0.000 | |
| SNU878 | | 0.239 | | | 0.250 | | | | 0.000 | | | 0.000 | | | 0.000 | | | 0.000 | | | 0.000 | | | | 0.000 | | | | 0.000 | |
| PLCPRF5 | | 0.287 | | | 0.000 | | | | 0.000 | | | 0.000 | | | 0.000 | | | 0.290 | | | 0.000 | | | | 0.000 | | | | 0.000 | |
| JHH5 | | 0.660 | | | 0.000 | | | | 0.670 | | | 0.000 | | | 0.000 | | | 0.000 | | | 0.000 | | | | 0.000 | | | | 0.000 | |
| JHH4 | | 0.986 | | | 0.570 | | | | 0.000 | | | 0.000 | | | 0.000 | | | 0.000 | | | 0.000 | | | | 0.590 | | | | 0.000 | |
| SNU423 | | 1.084 | | | 0.770 | | | | 0.390 | | | 0.000 | | | 0.000 | | | 0.000 | | | 0.210 | | | | 0.000 | | | | 0.000 | |
| Huh7 | | 2.154 | | | 1.370 | | | | 1.290 | | | 0.000 | | | 0.000 | | | 0.240 | | | 0.300 | | | | 0.000 | | | | 0.070 | |
| SNU387 | | 2.488 | | | 2.210 | | | | 0.990 | | | 0.000 | | | 0.000 | | | 0.000 | | | 0.000 | | | | 0.000 | | | | 0.100 | |
| SNU886 | | 3.350 | | | 2.160 | | | | 2.550 | | | 0.000 | | | 0.000 | | | 0.190 | | | 0.990 | | | | 0.000 | | | | 0.000 | |
| SNU761 | | 4.680 | | | 1.380 | | | | 4.540 | | | 0.000 | | | 0.000 | | | 0.260 | | | 0.800 | | | | 0.000 | | | | 0.000 | |
| HEP3B217 | | 4.758 | | | 4.080 | | | | 3.010 | | | 0.000 | | | 0.000 | | | 0.690 | | | 1.840 | | | | 0.000 | | | | 0.000 | |
| JHH2 | | 5.126 | | | 4.420 | | | | 3.650 | | | 0.000 | | | 0.000 | | | 0.720 | | | 1.740 | | | | 0.000 | | | | 0.080 | |
| JHH6 | | 5.391 | | | 5.330 | | | | 0.000 | | | 0.610 | | | 0.000 | | | 0.200 | | | 1.420 | | | | 0.000 | | | | 0.070 | |
| Huh1 | | 5.630 | | | 4.930 | | | | 4.320 | | | 0.000 | | | 0.000 | | | 0.000 | | | 1.170 | | | | 0.000 | | | | 0.120 | |
| **Median** | 0.263 | | | 0.120 | | | | 0.140 | | | 0.000 | | | 0.000 | | | 0.000 | | | 0.000 | | | | 0.000 | | | | 0.000 | |  |
| **Range** | 0.12-5.63 | | | 0-5.33 | | | | 0-4.54 | | | 0-0.61 | | | 0-0 | | | 0-0.72 | | | 0-1.84 | | | | 0-0.59 | | | | 0-0.12 | |  |

**Note:** RNA-seq data were extracted from the DepMap website (https://depmap.org/portal/download/). The relative expression values of *PRSS3* and its transcript variants were normalized with transcripts per million **(**TPM**)** shown as a log2 (TPM+1) scale. The expression values above the median are highlighted in gray.

# Table S2 Expression of *PRSS3* transcripts in 50 matched pairs of primary liver tumors and solid normal tissues

| Sample ID | *PRSS3* | | *PRSS3-V1* | | | *PRSS3-V2* | | | *PRSS3-V3* | | |  |
| --- | --- | --- | --- | --- | --- | --- | --- | --- | --- | --- | --- | --- |
|  | Normal | Tumor | | Normal | Tumor | | Normal | Tumor | | Normal | Tumor | |
| TCGA-DD-A1EB-01A | 0.55 | 0.18 | | 0.00 | 0.00 | | 0.55 | 0.18 | | 0.00 | 0.00 | |
| TCGA-DD-A3A6-01A | 0.76 | 0.14 | | 0.00 | 0.00 | | 0.75 | 0.14 | | 0.00 | 0.00 | |
| TCGA-BC-A110-01A | 1.16 | 9.40 | | 0.33 | 0.00 | | 0.83 | 9.40 | | 0.00 | 0.00 | |
| TCGA-DD-A1EH-01A | 1.19 | 49.80 | | 0.00 | 28.41 | | 1.19 | 21.05 | | 0.00 | 0.29 | |
| TCGA-DD-A114-01A | 1.42 | 0.38 | | 0.00 | 0.00 | | 1.42 | 0.38 | | 0.00 | 0.00 | |
| TCGA-DD-A1EJ-01A | 1.48 | 4.03 | | 0.14 | 0.13 | | 1.34 | 3.90 | | 0.00 | 0.00 | |
| TCGA-BC-A10Q-01A | 1.76 | 0.15 | | 0.00 | 0.00 | | 1.76 | 0.15 | | 0.00 | 0.00 | |
| TCGA-DD-A11A-01A | 1.78 | 5.61 | | 0.52 | 3.51 | | 1.26 | 2.10 | | 0.00 | 0.00 | |
| TCGA-FV-A23B-01A | 1.87 | 16.20 | | 0.00 | 8.92 | | 1.87 | 7.30 | | 0.00 | 0.00 | |
| TCGA-BC-A10Y-01A | 1.89 | 76.00 | | 0.21 | 54.59 | | 1.68 | 21.24 | | 0.00 | 0.18 | |
| TCGA-DD-A3A5-01A | 1.93 | 0.00 | | 0.56 | 0.00 | | 1.37 | 0.00 | | 0.00 | 0.00 | |
| TCGA-FV-A3I1-01A | 1.95 | 0.00 | | 0.21 | 0.00 | | 1.74 | 0.00 | | 0.00 | 0.00 | |
| TCGA-FV-A3I0-01A | 2.02 | 3.18 | | 0.00 | 0.00 | | 2.02 | 3.18 | | 0.00 | 0.00 | |
| TCGA-DD-A3A4-01A | 2.18 | 5.02 | | 0.00 | 0.00 | | 2.18 | 5.02 | | 0.00 | 0.00 | |
| TCGA-BD-A3EP-01A | 2.26 | 32.30 | | 0.23 | 22.55 | | 2.04 | 9.77 | | 0.00 | 0.00 | |
| TCGA-DD-A39X-01A | 2.28 | 28.10 | | 0.00 | 10.36 | | 2.28 | 17.17 | | 0.00 | 0.53 | |
| TCGA-DD-A11C-01A | 2.30 | 18.50 | | 0.45 | 15.79 | | 1.85 | 2.62 | | 0.00 | 0.08 | |
| TCGA-DD-A3A2-01A | 2.37 | 8.01 | | 0.18 | 3.73 | | 2.19 | 4.27 | | 0.00 | 0.00 | |
| TCGA-BC-A10Z-01A | 2.52 | 41.20 | | 0.00 | 17.70 | | 2.52 | 23.30 | | 0.00 | 0.18 | |
| TCGA-BC-A10X-01A | 2.55 | 1.64 | | 0.00 | 0.00 | | 2.55 | 1.64 | | 0.00 | 0.00 | |
| TCGA-BC-A10R-01A | 2.62 | 1.48 | | 0.00 | 0.00 | | 2.62 | 1.48 | | 0.00 | 0.00 | |
| TCGA-FV-A3R2-01A | 2.72 | 27.50 | | 0.00 | 25.74 | | 2.72 | 1.72 | | 0.00 | 0.00 | |
| TCGA-BC-A10T-01A | 2.83 | 2.09 | | 0.00 | 0.00 | | 2.83 | 2.09 | | 0.00 | 0.00 | |
| TCGA-DD-A118-01A | 2.89 | 0.00 | | 0.26 | 0.00 | | 2.63 | 0.00 | | 0.00 | 0.00 | |
| TCGA-G3-A3CH-01A | 3.23 | 8.10 | | 0.00 | 0.22 | | 3.23 | 7.88 | | 0.00 | 0.00 | |
| TCGA-DD-A1EL-01A | 3.48 | 0.24 | | 3.48 | 0.00 | | 0.00 | 0.24 | | 0.00 | 0.00 | |
| TCGA-EP-A3RK-01A | 4.13 | 0.26 | | 0.00 | 0.00 | | 4.13 | 0.26 | | 0.00 | 0.00 | |
| TCGA-DD-A39W-01A | 4.19 | 0.00 | | 0.15 | 0.00 | | 4.04 | 0.00 | | 0.00 | 0.00 | |
| TCGA-BC-A10U-01A | 4.34 | 1.28 | | 0.00 | 0.00 | | 4.34 | 1.28 | | 0.00 | 0.00 | |
| TCGA-EP-A26S-01A | 4.46 | 0.03 | | 2.42 | 0.00 | | 2.04 | 0.03 | | 0.00 | 0.00 | |
| TCGA-DD-A3A3-01A | 4.55 | 14.80 | | 0.00 | 9.54 | | 4.55 | 5.24 | | 0.00 | 0.00 | |
| TCGA-BC-A10W-01A | 4.64 | 0.42 | | 0.00 | 0.00 | | 4.64 | 0.42 | | 0.00 | 0.00 | |
| TCGA-DD-A1EI-01A | 4.71 | 0.33 | | 0.15 | 0.00 | | 4.56 | 0.33 | | 0.00 | 0.00 | |
| TCGA-BD-A2L6-01A | 4.78 | 132.0 | | 0.20 | 74.44 | | 4.59 | 57.46 | | 0.00 | 0.00 | |
| TCGA-DD-A116-01A | 5.27 | 2.09 | | 0.17 | 0.00 | | 5.10 | 2.09 | | 0.00 | 0.00 | |
| TCGA-FV-A2QR-01A | 5.50 | 36.40 | | 0.61 | 23.78 | | 4.89 | 12.59 | | 0.00 | 0.00 | |
| TCGA-EP-A12J-01A | 5.64 | 0.00 | | 0.00 | 0.00 | | 5.64 | 0.00 | | 0.00 | 0.00 | |
| TCGA-DD-A39V-01A | 5.96 | 0.04 | | 0.39 | 0.00 | | 5.58 | 0.04 | | 0.00 | 0.00 | |
| TCGA-DD-A39Z-01A | 6.70 | 0.00 | | 0.00 | 0.00 | | 6.70 | 0.00 | | 0.00 | 0.00 | |
| TCGA-DD-A3A8-01A | 6.85 | 0.04 | | 4.86 | 0.00 | | 1.99 | 0.04 | | 0.00 | 0.00 | |
| TCGA-DD-A119-01A | 7.03 | 10.40 | | 0.00 | 0.39 | | 7.03 | 10.04 | | 0.00 | 0.00 | |
| TCGA-BC-A216-01A | 8.50 | 67.80 | | 0.32 | 37.51 | | 7.98 | 30.31 | | 0.19 | 0.00 | |
| TCGA-DD-A1EC-01A | 8.97 | 1.02 | | 6.58 | 0.00 | | 2.39 | 1.02 | | 0.00 | 0.00 | |
| TCGA-DD-A1EG-01A | 10.00 | 32.00 | | 6.47 | 18.55 | | 3.56 | 13.40 | | 0.00 | 0.08 | |
| TCGA-DD-A11B-01A | 10.30 | 22.30 | | 0.00 | 13.23 | | 10.31 | 9.08 | | 0.00 | 0.00 | |
| TCGA-DD-A113-01A | 10.80 | 7.60 | | 0.23 | 4.14 | | 10.55 | 3.46 | | 0.00 | 0.00 | |
| TCGA-DD-A11D-01A | 11.00 | 18.20 | | 6.29 | 0.00 | | 4.71 | 18.23 | | 0.00 | 0.00 | |
| TCGA-DD-A1EE-01A | 14.10 | 0.76 | | 5.59 | 0.00 | | 8.55 | 0.76 | | 0.00 | 0.00 | |
| TCGA-DD-A3A1-01A | 15.10 | 45.20 | | 8.07 | 25.43 | | 7.07 | 19.77 | | 0.00 | 0.00 | |
| TCGA-ES-A2HT-01A | 29.40 | 7.21 | | 18.96 | 4.01 | | 10.31 | 3.21 | | 0.09 | 0.00 | |
| Median | 3.36 | 3.61 | | 0.15 | 0.00 | | 2.62 | 2.09 | | 0.00 | 0.00 | |
| Range | 0.55-29.40 | 0-132.0 | | 0-18.96 | 0-74.44 | | 0-10.55 | 0-57.46 | | 0-0.19 | 0-0.53 | |

**Note:** RNA-seq data were extracted from the FIREHOSE Broad GDAC (http://gdac.broadinstitute.org). The relative expression values of *PRSS3* and its transcript variants were normalized with TPM. The expression values above the median are highlighted in gray. The two expression values closest to the median are in red.

# Table S3 Expression predominance of *PRSS3* transcripts in HCC tumor tissues

| **Transcript variant** | Cutoff value (TPM) | | ***PRSS3^High^V^high^*** | | ***PRSS3^High^V^Low^*** | | ***PRSS3^Low^V^Low^*** | | ***PRSS3^Low^V^High^*** | |
| --- | --- | --- | --- | --- | --- | --- | --- | --- | --- | --- |
|  | Median | Range | n | % | n | % | n | % | n | % |
| ***PRSS3-V1*** | 0.00 | 0.00 -181.29 | 163 | 88.59 | 21 | 11.41 | 166 | 88.77 | 21 | 11.23 |
| ***PRSS3-V2*** | 1.39 | 0.00 - 704.01 | 173 | **94.02** | 11 | 5.98 | 175 | 93.58 | 12 | 6.42 |
| ***PRSS3-V3*** | 0.00 | 0.00 -1.23 | 48 | 26.09 | 136 | 73.91 | 185 | **98.93** | 2 | 1.07 |

**Note:** The HCC tumor samples were classified into the following groups in accordance with the expression of *PRSS3-SVs* (median expression level as cutoff value, **Table 1**): ***PRSS3^Hig^V^High^***: Highly expressed *PRSS3-SVs* in *PRSS3^High^* tumors; ***PRSS3^High^V^Low^***: Lowly expressed *PRSS3-SVs* in *PRSS3^High^* tumors; ***PRSS3^low^V^high^***: Highly expressed *PRSS3-SVs* in *PRSS3^Low^* tumors; ***PRSS3^Low^V^Low^***: Lowly expressed *PRSS3-SVs* in *PRSS3^Low^* tumors.

# Table S4. The CpG sites selected in the study and their genomic location in *PRSS3*

# Table S5. Spearman correlation analysis of the correlation between the expression of *PRSS3* transcripts and CG site methylation in human HCC cell lines

Cell lines^¶^: The number of cell lines with CpG site methylation data

RHO^¶¶:^ Spearman rank correlation coefficient or Spearman coefficient of rank correlation

# Table S6 Information on the OmicsLinkTM Expression clones of *PRSS3* transcripts

| **Plasmids** | **Catalog No** | **Accession No.** |
| --- | --- | --- |
| *PRSS3-V2* | EX-F0190-M35 | NM_002771.3 |
| *PRSS3-V3* | EX-A3595-M35 | NM_001197097.2 |
| *PRSS3-V4* | EX-Z9306-M35 | NM_001197098.1 |
| Vector control (pEZ-M35) | EX-NEG-M35 |  |

# Table S7 Coexpression of *PRSS3* transcript variants with associated hub genes in human HCC cell lines

| **Cell Line** | ***PRSS3*** | ***PRSS3-V1*** | ***PRSS3-V2*** | ***F2RL1*** | ***SMPDL3B*** | ***SLC43A3*** | ***DUOX2*** | ***TMEM45A*** | ***VNN1*** | ***GLUL*** | ***NKD1*** |
| --- | --- | --- | --- | --- | --- | --- | --- | --- | --- | --- | --- |
| **JHH1** | 0.04 | 0.03 | 0.00 | 5.79 | 2.66 | **4.22** | 0.01 | 0.30 | 0.01 | 8.06 | 0.83 |
| **SNU398** | 0.06 | 0.00 | 0.00 | 0.76 | 3.22 | 1.66 | 0.00 | 1.77 | 0.01 | **7.14** | 2.35 |
| **SNU475** | 0.07 | 0.00 | 0.00 | 2.50 | 0.77 | 1.54 | 0.08 | **3.17** | 0.01 | 6.65 | 0.19 |
| **HEPG2** | 0.07 | 0.00 | 0.00 | 2.76 | 0.70 | 0.12 | 0.12 | 3.82 | 0.76 | 8.68 | 5.96 |
| **LI7** | 0.08 | 0.00 | 0.16 | 5.50 | **1.29** | 0.32 | 0.20 | 1.40 | **0.39** | 7.79 | 0.04 |
| **SNU449** | 0.11 | 0.00 | 0.00 | 1.09 | 3.39 | 3.68 | 0.00 | 2.88 | 0.06 | 6.82 | 0.01 |
| **SKHEP1** | 0.12 | 0.12 | 0.00 | 1.52 | 0.37 | 5.80 | 0.10 | 2.65 | 2.26 | 5.87 | 0.08 |
| **HUH6** | 0.12 | 0.00 | 0.08 | 4.64 | 4.98 | 3.62 | 0.11 | **3.05** | 0.01 | 7.71 | 6.02 |
| **JHH7** | 0.16 | 0.00 | 0.21 | 3.86 | 1.87 | 1.16 | **0.04** | 1.23 | 0.03 | 8.54 | 2.08 |
| **HLF** | 0.18 | 0.00 | 0.18 | 6.04 | 0.26 | 5.22 | 0.00 | 3.59 | 0.07 | 3.87 | 0.08 |
| **SNU182** | 0.23 | 0.12 | 0.12 | 5.57 | 0.12 | 1.73 | 0.07 | 4.14 | 0.11 | 8.88 | 1.48 |
| **SNU878** | 0.24 | 0.25 | 0.00 | 3.26 | 3.15 | 4.99 | 0.20 | 2.58 | 5.52 | 8.85 | 0.51 |
| **PLCPRF5** | 0.29 | 0.00 | 0.00 | 3.55 | 2.74 | 0.34 | 0.03 | 4.55 | 0.70 | 6.67 | **0.26** |
| **JHH5** | 0.66 | 0.00 | 0.67 | **4.19** | 2.42 | 0.26 | **0.04** | 1.86 | 0.23 | 8.40 | 0.37 |
| **JHH4** | 0.99 | 0.57 | 0.00 | 6.79 | 2.86 | 5.73 | 0.03 | 1.12 | 0.30 | 6.17 | 0.01 |
| **SNU423** | 1.08 | 0.77 | 0.39 | 4.65 | 0.12 | 7.29 | 0.00 | 5.95 | 1.68 | 6.71 | 0.07 |
| **HUH7** | 2.15 | 1.37 | 1.29 | 2.48 | 3.72 | **4.46** | 0.34 | 4.56 | 0.26 | 8.69 | 0.96 |
| **SNU387** | 2.49 | 2.21 | 0.99 | 3.09 | 0.44 | 6.07 | 0.01 | 4.54 | 1.48 | 7.32 | **0.32** |
| **SNU886** | 3.35 | 2.16 | 2.55 | 5.41 | 0.52 | 5.58 | 0.12 | 2.99 | 3.48 | 5.97 | 0.11 |
| **SNU761** | 4.68 | 1.38 | 4.54 | 5.07 | 0.98 | 6.24 | 1.68 | 4.57 | 5.22 | 6.00 | 0.01 |
| **HEP3B217** | 4.76 | 4.08 | 3.01 | 2.83 | **1.80** | 7.71 | 0.00 | 3.20 | **0.36** | 7.88 | 2.97 |
| **JHH2** | 5.13 | 4.42 | 3.65 | 6.78 | 0.11 | 5.55 | 0.12 | 5.64 | 1.82 | 6.21 | 0.16 |
| **JHH6** | 5.39 | 5.33 | 0.00 | 5.49 | 0.19 | 7.69 | 0.01 | 4.31 | 1.46 | 5.65 | 1.32 |
| **HUH1** | 5.63 | 4.93 | 4.32 | **3.95** | 3.22 | 1.77 | 0.01 | 2.68 | 7.40 | **6.88** | 0.07 |
| **Mean** | 0.26 | 0.12 | 0.14 | 4.07 | 1.55 | 4.34 | 0.04 | 3.11 | 0.37 | 7.01 | 0.29 |
| **Range (Min)** | 0.04 | 0.00 | 0.00 | 1.52 | 0.11 | 0.12 | 0.00 | 0.30 | 0.01 | 3.87 | 0.01 |
| **Range (Max** | 5.63 | 5.33 | 4.54 | 3.95 | 4.98 | 7.71 | 1.68 | 5.95 | 7.40 | 8.88 | 6.02 |

**Note:** The data information is shown in **Table S1**. The expression values above the median are highlighted in gray.

# Table S8. Coexpression significance of the hub genes with *PRSS3* in human HCC cell lines

| **Gene** | **Pearson** | **Spearman** | ***P* value** |
| --- | --- | --- | --- |
| ***F2RL1*** | 0.265 | 0.274 | 0.211 |
| ***SMPDL3B*** | -0.219 | -0.224 | 0.305 |
| ***DUOX2*** | 0.296 | 0.024 | 0.160 |
| ***SLC43A3*** | 0.509 | 0.589 | 0.011 |
| ***TMEM45A*** | 0.407 | 0.502 | 0.049 |
| ***VNN1*** | 0.543 | 0.660 | 0.006 |
| ***GLUL*** | -0.280 | -0.273 | 0.186 |
| ***NKD1*** | -0.165 | -0.199 | 0.440 |

**Note:** The information was obtained from the DepMap website (https://depmap.org/portal/download/).

# Supplementary Figures

**
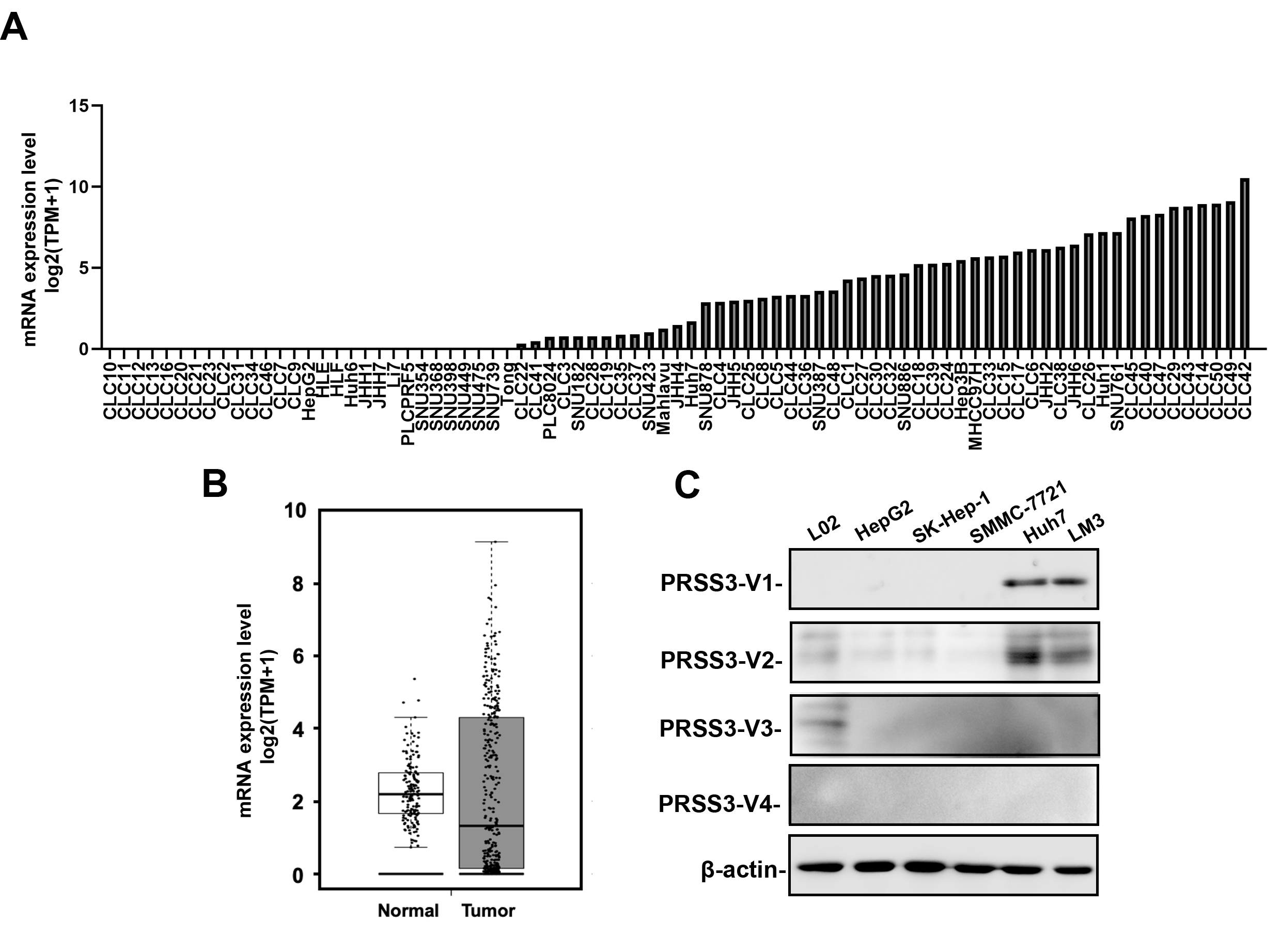
**

**Figure S1.** *PRSS3* expression in human HCC cell lines and tissues. **(A)** The mRNA expression level of *PRSS3* in 81 liver cancer cell lines. Data were extracted from the Cancer Model Repository (LIMORE, https://www.picb.ac.cn/limore/home). **(B)** GEPIA portal analysis of TCGA and GETx RNA-seq results of *PRSS3* mRNA in HCC (tumor = 369) and normal liver tissues (normal = 160) (*p* value cutoff: 0.01). Graphs were modified from the GEPIA website (<http://gepia.cancer-pku.cn>). **(C)** Western blot analysis of PRSS3 isoforms using noncommercial, custom antibodies against PRSS3-V1~-V4.

**
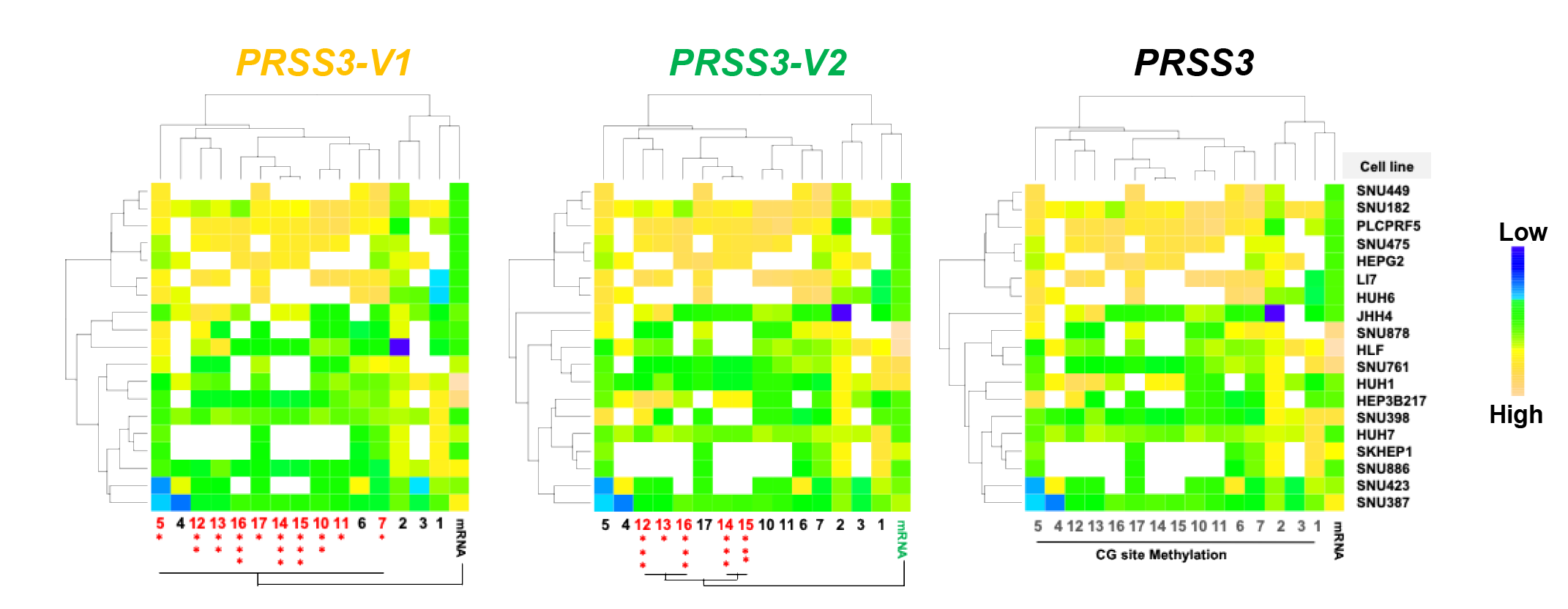
**

**Figure S2.** Clustered heatmap showing the correlation between CpG site methylation and *PRSS3* transcript expression. Data were visualized by using correlation as a distance function for heatmap cluster analysis of the correlation between the expression and CpG site methylation of *PRSS3* in HCC cell lines. The statistical significance of correlation coefficients between CpG sites and mRNA expression of PRSS3 transcripts are shown with red asterisks at the bottom (**Table S5**).

**
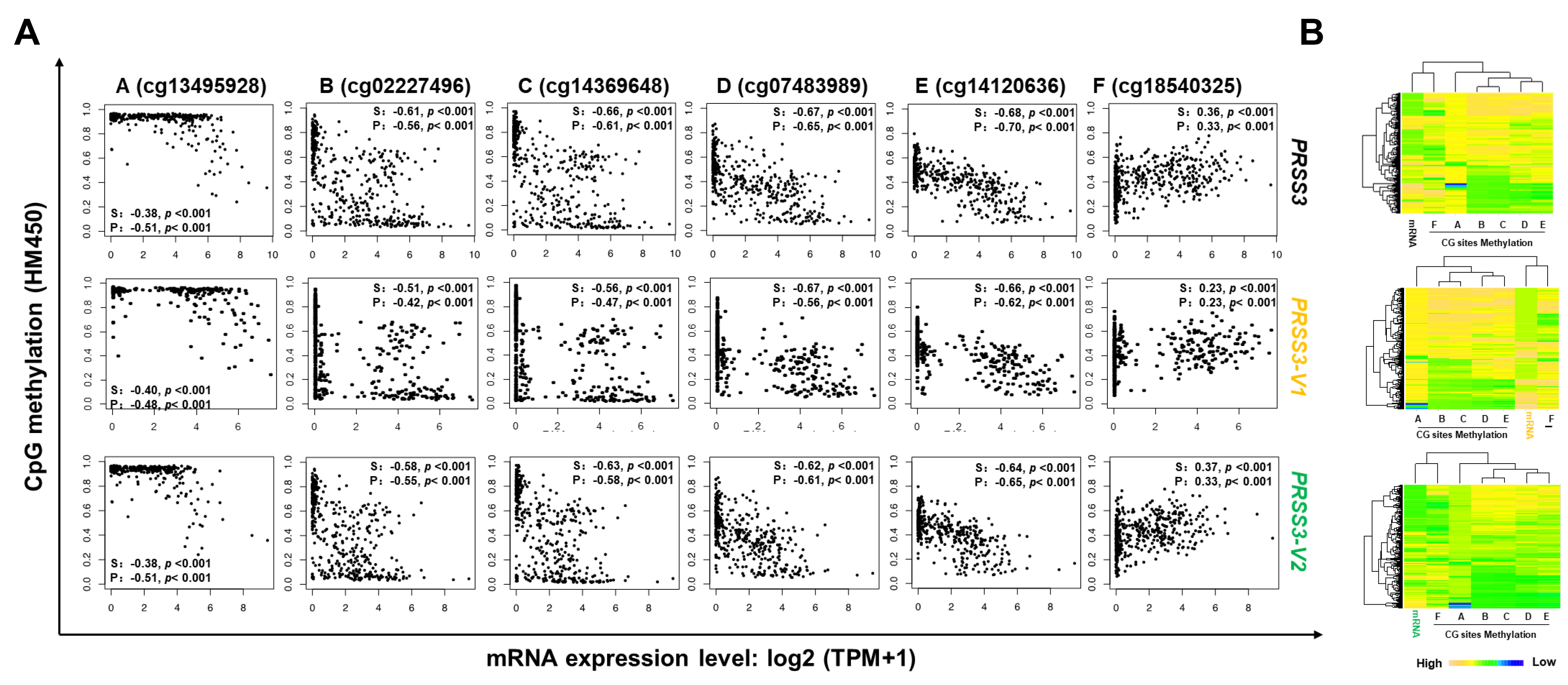
**

**Figure S3.** Association of CpG site methylation with the mRNA expression level of *PRSS3* transcripts. (A) Spearman and Pearson correlation analysis of PRSS3 mRNA expression associated with intragenic CpG site methylation in human primary liver tumor samples (n=414). (B) Clustering analysis of intragenic CpG site methylation with *PRSS3* transcript levels in HCC tissue specimens.

**
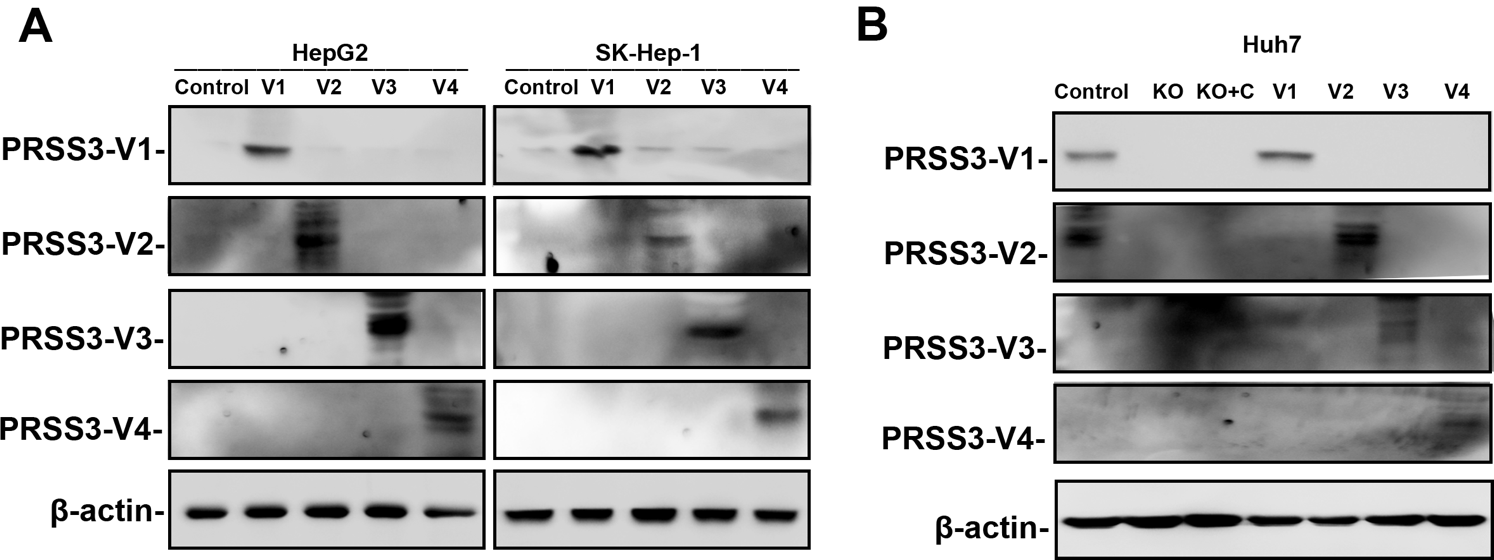
**

**Figure S4.** Western blot validating the ectopic expression of PRSS3 isoforms in HepG2 and SK-Hep-1 cells **(A)** and Huh7 cells **(B)**.


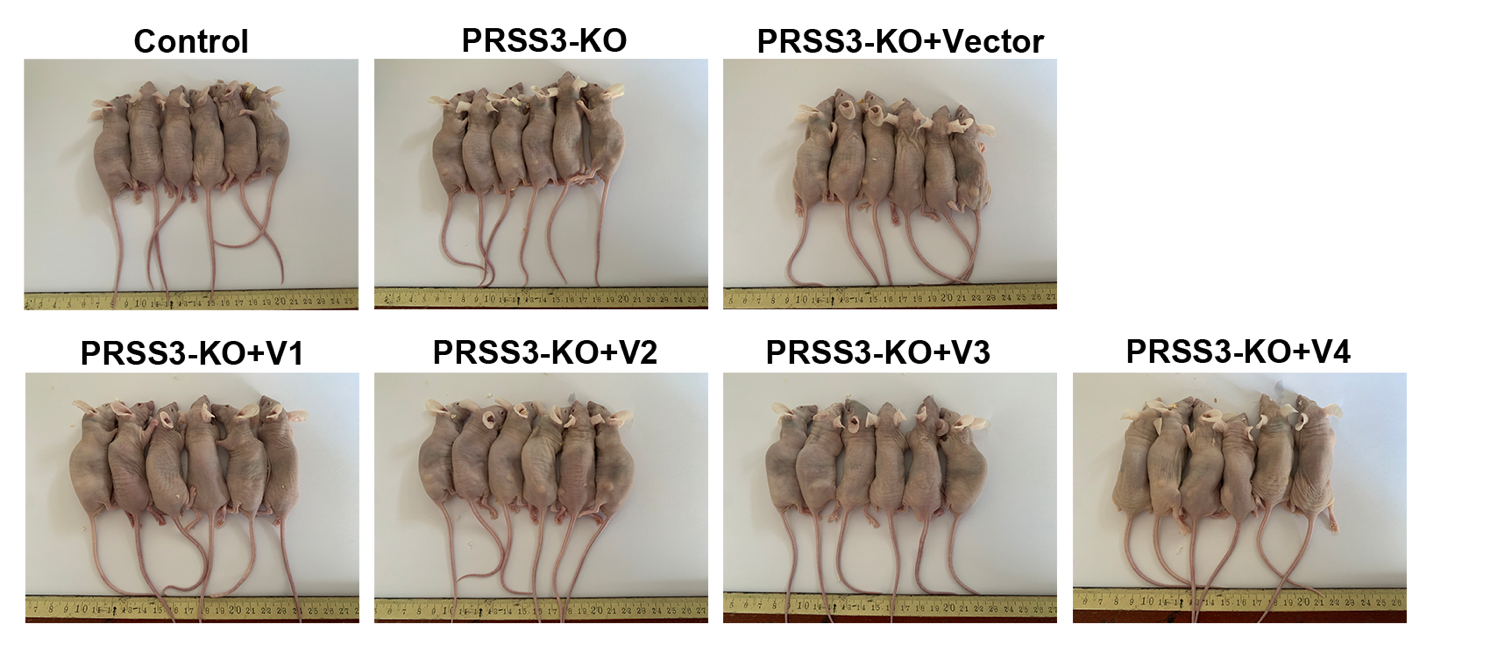


**Figure S5.** Effects of the *PRSS-SVs* on HCC tumorigenicity using the *PRSS3 ^KO+V^* cell model. Photographs showing the different groups of xenograft tumor-bearing mice sacrificed on day 15.


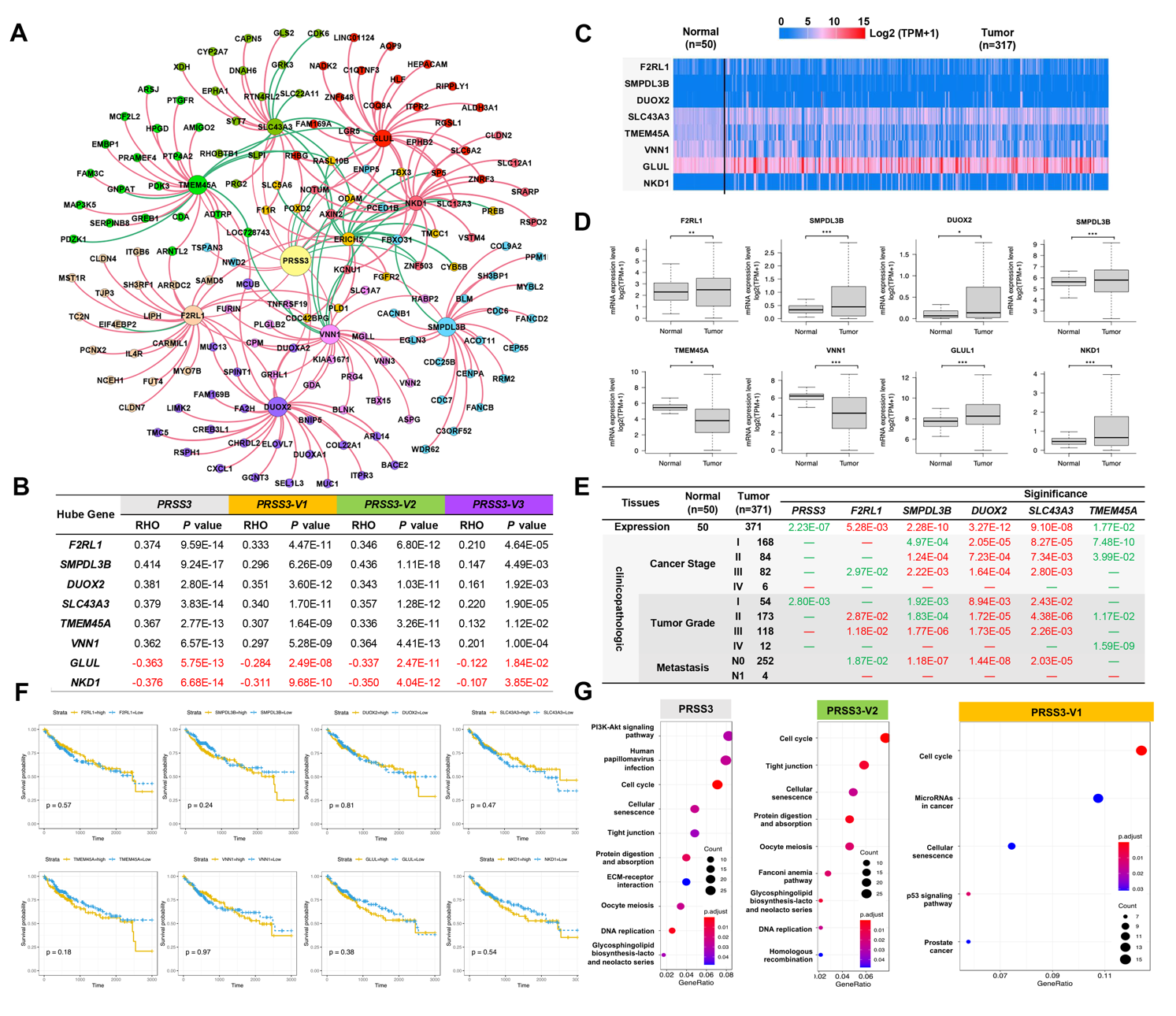


**Figure S6.** *PRSS3* transcript-associated genes and pathways in HCC. **(A)** Directed network analysis of the PRSS3 downstream target genes using network analysis of a dataset available from SEEK (http://seek.princeton.edu). Eight genes most associated with *PRSS3* transcripts in the network were selected as the key hub genes. **(B)** Significance of the coexpression of *PRSS3* transcripts and the key hub genes in human HCC using RHO (Spearman rank correlation coefficient or Spearman coefficient of rank correlation). Hub genes positively (black font) or negatively (red front) associated with *PRSS3* transcript variants were profiled. **(C)** Heatmap visualization of the expression of the hub genes in TCGA-LIHC tissues. Data were obtained from UALCAN (https://www.ualcan.path.uab.edu/). **(D)** Expression of the panel of hub genes in 371 liver tumor samples compared to 50 normal tissues. Data were obtained from the FIREHOSE Broad GDAC (http://firebrowse.org/). **(E)** The expression of the hub genes correlating to clinicopathologic features in HCC patients. Significance: upregulation (red font), downregulation (green front); “-” no significance; ND: no data. **(F)** Log rank test of overall survival of HCC patients in high and low expression groups that were clustered based the cutoff value of the median mRNA expression level of the genes in the samples. **(G)** KEGG pathway enrichment of the hub genes in association with PRSS3, PRSS3-V1 and PRSS3-V2 (p-adjusted <0.05; cluster Proﬁler).
